# Spatial early warning signals of social and epidemiological tipping points in a coupled behaviour-disease network

**DOI:** 10.1038/s41598-020-63849-0

**Published:** 2020-05-06

**Authors:** Brendon Phillips, Madhur Anand, Chris T. Bauch

**Affiliations:** 1 University of Waterloo, Department of Mathematics, Waterloo, N2L 3G1 Canada; 2 University of Guelph, School of Environmental Sciences, Guelph, N1G 2W1 Canada

**Keywords:** Applied mathematics, Computational models, Viral infection

## Abstract

The resurgence of infectious diseases due to vaccine refusal has highlighted the role of interactions between disease dynamics and the spread of vaccine opinion on social networks. Shifts between disease elimination and outbreak regimes often occur through tipping points. It is known that tipping points can be predicted by early warning signals (EWS) based on characteristic dynamics near the critical transition, but the study of EWS in coupled behaviour-disease networks has received little attention. Here, we test several EWS indicators measuring spatial coherence and autocorrelation for their ability to predict a critical transition corresponding to disease outbreaks and vaccine refusal in a multiplex network model. The model couples paediatric infectious disease spread through a contact network to binary opinion dynamics of vaccine opinion on a social network. Through change point detection, we find that mutual information and join count indicators provided the best EWS. We also show the paediatric infectious disease natural history generates a discrepancy between population-level vaccine opinions and vaccine immunity status, such that transitions in the social network may occur before epidemiological transitions. These results suggest that monitoring social media for EWS of paediatric infectious disease outbreaks using these spatial indicators could be successful.

## Introduction

Resurgences of vaccine-preventable diseases severely stress public health systems, interrupt tourism and public services, and disrupt economies through the huge costs of large-scale interventions^1^. These impacts motivate the study of factors that support vaccine uptake. Undervaccination may be attributed to vaccine refusal^2^, the spread of anti-vaccine opinion facilitated by media coverage and its sensationalisation of true adverse vaccine effects^3^, the expectation of adverse effects^4^, misstatement of the cause of illnesses^5^, the spread of other rumours and false information^6^, and the effect of social norms^7^.

These phenomena show how the social diffusion of information is heavily responsible for the trajectory of disease spread through its ability to alter individual behaviour. Much work has modelled opinion dynamics for different applications through the use of voter models^8^ and majority opinion models^9^, among other frameworks, and their combination with network structure has revealed much about the occurrence of opinion cascades^10^ and forecasting^11^. For instance, models coupling behavioural dynamics and spreading processes change the predicted dynamics of influenza transmission^12^ and climate change^13^ alike.

Opinion propagation can be represented by information diffusion through social networks^14^. Similarly, infection spread is often conceptualized as spreading through a physical contact network^15^. Many models explore the dynamics of $n$-layer multiplex networks, where each layer represents a different aspect of the dynamics of a single coupled system^16–19^. In these cases, the theory of phase transitions in spatially structured systems is important. For instance, epidemic regimes have previously been modelled as the outcome of phase transitions in physical systems and are featured widely in the epidemiology literature^20–26^. Generally, phase transitions occur when a physical system moves from one state to another after going through some critical point. Second-order transitions occur when a macroscopic variable varies continuously^27^ and are called critical transitions^28^.

Systems approaching these critical transitions sometimes display characteristic spatial or temporal behaviours called early warning signals (EWS) that can predict coming epidemic disease outbreaks and other events^29^. EWS are perhaps better defined as statistically significant, recognisable and characteristic behaviours known to precede critical transition in dynamical systems^30,31^. For instance, critical slowing down can precede both first- and second-order transitions^31,32^ and is accompanied by the divergence of correlation length in a physical system^33^.

Many statistics have been used to study EWS in spatially extended systems; temporal^34,35^ and spatial correlation^36,37^ have been found to precede transitions in spreading processes. Other measurements have been applied to spin systems, where each site in a lattice may be in one of two possible states, possibly partially dependent on the state of neighbouring sites. The spin model has also been applied to opinion dynamics; a simple voter model with binary opinion dynamics is analogous to a physical spin system, where particles represent agents and spins represent different opinions^38^. Consensus formation can be seen as a second-order phase transition to an ordered state (where all spins are aligned). In this regime, knowledge of the opinion of a single agent predicts the opinion of all other agents in the system^39^. Since the transition in finite networks is smooth^40^, the distance across the network over which the opinions of connected agents are strongly correlated increases smoothly; this is analogous to divergence of the correlation length of a physical system^41^.

Above some critical temperature, disordered systems take the form of a spin glass. In a spatial opinion model, this describes a state where opinions between neighbours are generally uncorrelated^42^. On a static network, this state induces a larger number of edges between dissimilar neighbours as compared to that of consensus regimes. This is related to join count statistics, where the numbers of edges between like neighbours are compared to the number between dislike neighbours as a test of geographical distribution. This is arguably the most natural and well-defined measure for graphs presenting binary data and is used for spatial analysis^43^.

The necessity of disease surveillance and early warning signals for outbreaks has been discussed in multiple contexts, from epidemic mitigation to bioterrorism prevention^44–46^. Potential mitigation of unnecessary expense motivates us to find reliable EWS that remain easily computable on large high-resolution data sets. Furthermore, the study of EWS in coupled disease-behaviour multiplex networks has received relatively little attention, suggesting a significant gap in the literature. Our objective is to evaluate and compare the relative merits of the mutual information, Moran’s I, Geary’s C and join count statistics as EWS of the occurrence of epidemics and changes in aggregate opinion on a coupled disease-behaviour network model. We use three differently parametrised models (V1, V2 and V3) coupling a binary vaccination opinion dynamic to an SIRV epidemic process. The resulting trends in the EWS for model V2 will be explored in the Results and Discussion sections, with V1 and V3 presented in Supplementary Information S5.

The outline of this paper is as follows: the Methods section will present the EWS and their derivations and give the details of the model used. The Results section will analyse the trends in the warning signals and the Discussion section will present a review of the study and any shortcomings of our approach, with further results pertinent to the study presented in the Supplementary Information.

## Methods

We assume an acute, self-limiting infection that confers lifelong natural immunity upon recovery, and for which a vaccine is readily available. Similar premises have been used to represent the natural history of many paediatric infectious diseases such as measles^47^. In particular, we assume an $SIR{V}_{p}$ natural history consisting of four mutually exclusive disease states. Agents are initially susceptible to infection ($S$). Upon infection the agent enters the infected state ($S\to I$), which we treat as a combination of both the latent, ill and infectious periods^48^. Upon clearing the infection, agents enter the recovered state of lifelong immunity ($I\to R$); additionally, susceptible agents may be vaccinated and so enter the vaccinated state ($S\to {V}_{p}$)^49^.

We also include injunctive social norms (i.e. peer pressure) as well as a perceived cost of vaccination that captures both economic costs and the fear of perceived adverse vaccine effects^50^. As in some models^36^, we include a noise parameter $\xi$ to account for environmental and demographic stochasticity^51^ with the simplifying assumption of perfect vaccination^52^ (reversion from the recovered state to the susceptible only through agent death). During simulation, each time step represents a single week.

### The model

We model a multilayer network where each layer is given an identical undirected Erdös-Rényi random graph with size $N$ and mean node degree $\langle {Q}_{n}\rangle$. Each agent $n$ can be described by a pair of states; for instance, each agent is assigned the joint state $({V}_{s},{V}_{p})$ at the start of the simulation with probability $\alpha$ (they are a pro-vaccine vaccinated agent), else they are initialised with joint state $(N,S)$ (an anti-vaccine susceptible agent) with probability $1-\alpha$.

**Figure 1:**
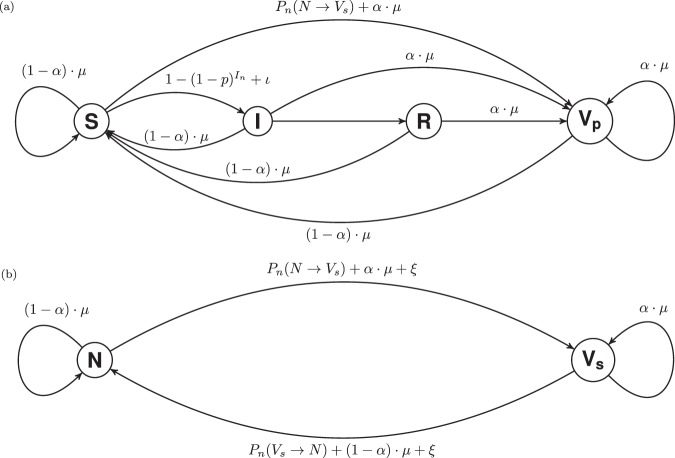
Representation of the infection **(a)** and opinion **(b)** dynamics of the model occurring on the physical and social layers of the 2-layer network, respectively. **(a)** Effective contacts occur between susceptible $S$ and infected $I$ agents with probability $p$ per time step ($1$ week). Upon deciding to vaccinate (with probability ${{\mathbb{P}}}_{n}(N\to {V}_{s})$), a susceptible agent $n$ becomes physically vaccinated ($S\to {V}_{p}$). Infection lasts $\ell =2$ weeks after which agents recover ($I\to R$). Upon death (with probability $\mu$ per week), an agent is “rebirthed” with either vaccinated (probability $\alpha \cdot \mu$) or susceptible (probability $\mathrm{(1}-\alpha )\cdot \mu$) status. **(b)** Per time step, each agent switches between pro- (${V}_{s}$) and anti-vaccine ($N$) opinion with probabilities ${{\mathbb{P}}}_{n}(N\to {V}_{s})$ and ${{\mathbb{P}}}_{n}({V}_{s}\to N)$ respectively upon interaction with a dissenting neighbour. $\alpha$ gives the probability of being birthed with pro-vaccine opinion ${V}_{s}$.

The social process follows an $N{V}_{s}$ dynamic (Fig. 1b), representing pro- (${V}_{s}$) and anti-vaccine ($N$) opinion for each agent $n$. $\xi$ represents the probability of any agent switching opinion randomly in each week and ${{\mathbb{P}}}_{n}(N\to {V}_{s})$ represents the probability of switching from anti-vaccine opinion to pro-vaccine opinion ($N\to {V}_{s}$) upon interaction with a disagreeing neighbour. We introduce an imitation dynamic by having each agent $n$ compare its opinion with a single randomly chosen social contact (a neighbouring agent on the social layer) each week; $n$ then changes its vaccination opinion only if there is disagreement (the agent and the neighbour have different vaccine opinions). This change of opinion depends on the perceived risk of vaccine adverse effects $\kappa$ (*“vaccine risk”*) and ${I}_{n}$ (the number of infected physical neighbours of $n$) according to the rules

1 $${{\mathbb{P}}}_{n}(N\to {V}_{s})=\frac{1}{1+{\rm{e}}{\rm{x}}{\rm{p}}(\,-\,\Delta {U}_{n}^{N\to {V}_{s}})},\,{{\mathbb{P}}}_{n}({V}_{s}\to N)=\frac{1}{1+{\rm{e}}{\rm{x}}{\rm{p}}(\,-\,\Delta {U}_{n}^{{V}_{s}\to N})},$$

where the indices $\Delta {U}_{n}^{N\to {V}_{s}}$ and $\Delta {U}_{n}^{{V}_{s}\to N}$ in Eq. (1) are utility functions defined as

2 $$\Delta {U}_{n}^{N\to {V}_{s}}=-\,\sigma \left(\frac{{Q}_{n}^{N}-{Q}_{n}^{{V}_{s}}}{{Q}_{n}}\right)-(\kappa -{I}_{n}),\,\Delta {U}_{n}^{{V}_{s}\to N}=-\,\sigma \left(\frac{{Q}_{n}^{{V}_{s}}-{Q}_{n}^{N}}{{Q}_{n}}\right)+(\kappa -{I}_{n})\mathrm{}.$$

Here, $\sigma$ represents the strength of an injunctive social norm to maintain the current opinion (referred to as *“social norm”*), while ${Q}_{n}^{\ast }$ represents the number of neighbours of $n$ with opinion $\ast$ and ${Q}_{n}$ represents the total number of social contacts of $n$.

The epidemiological dynamics (which we term ‘physical dynamics’) follow an $SIR{V}_{p}$ process (Fig. 1a), in which an agent $n$ can progress through each of four disease compartments: $S$ (susceptible), $I$ (infected), $R$ (recovered) and ${V}_{p}$ (vaccinated). Every week (i.e. in each time step), each susceptible agent $n$ interacts with all its physical neighbours; each effective interaction carries the probability $p$ of infection ($S\to I$), so that each susceptible agent faces the total probability $1-{\mathrm{(1}-p)}^{{I}_{n}}$ of infection in a single week. The duration of the illness is $\ell$ weeks (with no impact on mortality), after which $n$ gains lifelong natural immunity ($I\to R$).

Alternately, if a susceptible agent $n$ adopts a pro-vaccine opinion, they are immediately vaccinated $(S\to {V}_{p})$ and gain lifelong vaccine-derived immunity. We also assume that only susceptible agents are vaccinated. Thus, individual agents may change their opinion about vaccination multiple times in their life ($N\to {V}_{s}\to N$), but once they are vaccinated they may not become unvaccinated ($S\to {V}_{p}$). This in turn creates an asymmetry between disease dynamics and social dynamics that will have implications for the model predictions. We will discuss this in the Results section.

Each agent $n$ has probability $\mu$ of dying each week, upon which they are replaced by a new susceptible individual who is a pro-vaccine vaccinated agent $({V}_{s},{V}_{p})$ with probability $\alpha$, or an anti-vaccine susceptible agent $(N,S)$ with probability $1-\alpha$, keeping the same physical and social contacts as the agent they replaced (that is, the network is static). Case importation is accounted for by infecting a randomly selected proportion $\iota$ of susceptible agents at the start of each week, and noise is introduced to the model by changing the vaccine opinions of a randomly selected proportion $\xi$ of the entire population weekly.

At the start of the simulation, some susceptible agent is randomly selected as an index patient and infected; subsequent disease spread is governed solely by environment and inter-agent interaction. Models V1, V2 and V3 all use these model dynamics, and a flowchart and detailed written description of the model transitions are given in Supplementary Information S2. A complete list of the variables used is given in Supplementary Table S1.

### Early warning signals

*Mutual information* ${\mathscr{M}}$ is defined as

3 $${\mathscr{M}}({\mathscr{X}},{\mathscr{Y}})=\sum _{x\in {\mathscr{X}}}\sum _{y\in {\mathscr{Y}}}{\mathbb{P}}(x,y)\cdot {\log }_{2}\left(\frac{{\mathbb{P}}(x,y)}{{\mathbb{P}}(x)\cdot {\mathbb{P}}(y)}\right),$$

where $X$ and $Y$ are discrete random variables; $x$ takes value on the set ${\mathscr{X}}=\{{x}_{1},{x}_{2},{x}_{3},\ldots \}$ and $y$ on set ${\mathscr{Y}}=\{{y}_{1},{y}_{2},{y}_{3},\ldots \}$, with ${\mathbb{P}}$ a joint probability mass function of $X$ and $Y$^53^. Mutual information is an entropy-based quantification of the “shared information” of two random variables quantifying how knowledge of one decreases the uncertainty of the other and vice versa^54^. Mutual information peaks at the critical temperature of spin systems during second-order transitions and has been widely used in detecting phase transitions^55,56^; an advantage of this statistic is its ability to quantify non-linear dependence, unlike Moran’s I and covariance which only account for linear dependence.

*Join counts* quantify the degree of clustering by giving the number of adjacencies between agents of different types. We divide the population into two attributive classes, with ${V}_{s}$ the compartment of pro-vaccine agents and $N$ the compartment of anti-vaccine agents. Let [Ψ, Ω] be the number of social interactions between agents with vaccine opinions Ψ and Ω; then $[N,N]$ represents the number of nearest-neighbour interactions between anti-vaccine agents, $[{V}_{s},{V}_{s}]$ the number of interactions between pro-vaccine agents and $[N,{V}_{s}]$ the number of interactions between pro- and anti-vaccine agents. These can be written as

4 $$[N,{V}_{s}]=\frac{1}{2}\sum _{j,k}{\omega }_{jk}{({x}_{j}-{x}_{k})}^{2},\,[N,N]=\frac{1}{2}\sum _{j,k}{\omega }_{jk}(1-{x}_{j})(1-{x}_{k}),\,[{V}_{s},{V}_{s}]=\frac{1}{2}\sum _{j,k}{\omega }_{jk}{x}_{j}{x}_{k},$$

where ${\omega }_{j,k}=1$ if agents $j$ and *k* are social neighbours, with ${\omega }_{j,k}=0$ otherwise ($\omega$ is the adjacency matrix of the social network); ${x}_{n}$ represents the *opinion score* of agent $n$, defined as

5 $${x}_{n}=\left\{\begin{array}{cc}1 & k\in {V}_{s}\,({x}_{n}\,\text{has a pro} \mbox{-} \text{vaccine opinion})\\ 0 & \text{else}\end{array}\right..$$

In an opinion model, clustering manifests as agents consistently having a higher number of like-minded neighbours than expected based on the global prevalence of the opinion; join counts are then used to test the null hypothesis of positive correlation^57^. Join counts are used in many fields as a categorical test of spatial autocorrelation, including ecology^58^ and geographical information systems^59^. In all parameter realisations here, the number of joins are counted naïvely rather than calculated. Joins between like-minded agents (e.g. $[N,N]$ and $[{V}_{s},{V}_{s}]$ joins) will be called *similar joins*, and edges between disagreeing neighbours (e.g. $[N,{V}_{s}]$) will be called *dissimilar joins*.

The *Moran’s I* coefficient ${\mathscr{J}}$ quantifies spatial correlation and is defined

6 $${\mathscr{J}}=\frac{N}{W}\cdot \frac{\sum _{j,k}{\omega }_{jk}({x}_{j}-\overline{x})({x}_{k}-\overline{x})}{\sum _{j}{({x}_{j}-\overline{x})}^{2}},$$

where $W={\sum }_{j,k}{\omega }_{jk}$ and $\bar{x}=\frac{1}{N}{\sum }_{j}{x}_{j}$ represents the mean opinion score of the population^43^. Used as a global statistic, Moran’s I gives the degree of correlation between the values of neighbouring patches (agents and their social contacts); here, the numerical value of the vaccine opinion is the same as described in Eq. (5). Algebraic manipulation of Eq. (6) using Eq. (5) gives

7 $${\mathscr{J}}=\frac{N}{W}\cdot \frac{2\cdot [{V}_{s},{V}_{s}]-2\bar{x}\cdot (2\,[{V}_{s},{V}_{s}]+[N,{V}_{s}])+W\cdot {\bar{x}}^{2}}{(1-2\bar{x})\cdot [{V}_{s}]+N\cdot {\bar{x}}^{2}},$$

(full derivation given in Supplementary Information S4); we can then consider Moran’s I as a measure derived from the linear combination of join counts. Positive values signify spatial correlation, with negative values signifying anticorrelation.

The *Geary’s C* coefficient ${\mathscr{C}}$ is yet another measure of spatial correlation based on the cross-product (like Moran’s I)^43^, but unlike Moran’s I it accounts for the difference in opinion between two neighbours^60^. It is given as

8 $${\mathscr{C}}=\frac{N-1}{W}\frac{\sum _{j,k}{\omega }_{jk}{({x}_{j}-{x}_{k})}^{2}}{\sum _{j}{({x}_{j}-\bar{x})}^{2}}\mathrm{}.$$

Lower values show spatial correlation, and large values represent anticorrelation. Like Moran’s I (Eq. (7)), Geary’s C can also be expressed as a linear combination of join counts

9 $${\mathscr{C}}=\frac{N-1}{W}\frac{2\cdot [N,V]}{(1-2\bar{x})\cdot [V]+N\cdot {\bar{x}}^{2}};$$

this expression is derived in Supplementary Information S4.

### Parametrisation

The birth/death rate in the model was set at $\mu =2.4\times {10}^{-4}$, giving each agent a mean life expectancy of 80 years. The network size $N=40000$ was chosen to represent a small town where each agent *n* has effective physical contact with $\langle {Q}_{n}\rangle =30$ neighbours per week, where an effective contact is defined as any interaction between agents that allows for infection and/or the communication of opinion. The case importation proportion ratio $\iota =2.5\times {10}^{-5}$ was added to provide periodic impulses of infection as a test of resilience in endemic disease regimes. Here, an ensemble of $100$ simulations using parameters $\kappa =0$, $\sigma =0$ and $\alpha =0.05$ returned the values $\langle S\rangle ,\langle R\rangle  < 0.05$ at *equilibrium* (defined in Supplementary Information S3), where $\langle \Psi \rangle$ represents the mean number of agents with (social or physical) state $\Psi$, averaged over all realisations of that combination of parameter values.

**Figure 2:**
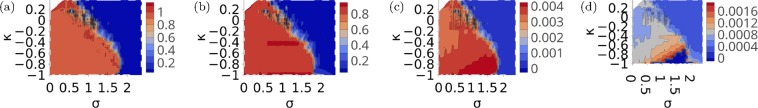
Contour plots of the region $(\sigma ,\kappa )\in [0,2.4]\times [-1,0.2]$ of the parameter plane, capturing the transition dynamics of both the social and physical dynamics averaged over 20 realisations of each set of parameters; $\sigma$ represents the strength of the social norm, and $\kappa$ the vaccine risk. **(a)** $\langle {V}_{s}\rangle$ (proportion of pro-vaccine agents), **(b)** $\langle {V}_{p}\rangle$ (vaccine coverage), and the corresponding trends in **(c)** $\langle {\mathscr{M}}\rangle$ (mutual information) and **(d)** $\langle N,{V}_{s}\rangle$ (dissimilar join count).

The infectivity $p=0.2$ was chosen to reflect the reproduction rate of a measles infection commonly estimated from empirical data^61^; effective contacts occur in the simulation once per week during the period of infection. The probability of randomly switching opinion ${\xi }_{1}=1\times {10}^{-4}$ was included as a source of noise. We found that the parameter ranges for vaccine risk $\kappa \in [-1,1]$ and social norm $\sigma \in [0,3]$ were sufficiently broad to capture transitions in both social and physical dynamics (Fig. 2a,b), as well as the corresponding trends in the mutual information (Fig. 2c) and dissimilar join count (Fig. 2d).

The contours in each panel of Fig. 2 show the obvious correspondence between transitions in the social and physical dynamics of the model, and substantial changes in $\langle {\mathscr{M}}\rangle$ and $\langle N,N\rangle$; here, the dissimilar join count $\langle N,{V}_{s}\rangle$ (Fig. 2d) and mutual information ${\mathscr{M}}$ (Fig. 2c) increase while the vaccine risk $\kappa$ and social norm $\sigma$ parameters increase towards their respective (pre-transition) threshold values. These trends are generally asymmetric about both transitions; this can be seen in Fig. 4, where post-transition trends do not exhibit similarly detectable warnings (if any). This Parametrisation applies to model V2. The corresponding parametrisations and contour plots of models V1 and V3, as well as their post-transition trends are presented through the Supplementary Information.

**Figure 3:**
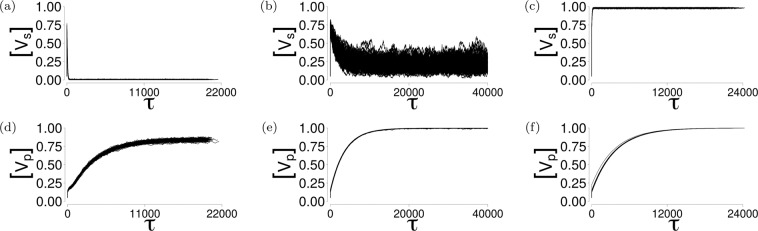
Time series demonstrating high sensitivity of the social dynamics to small changes (both positive and negative) in vaccine risk $\kappa$ when the strength of the social norm $\sigma =0$. All panels show the results of $100$ realisations of respective parameter combinations. **(a)** $[{V}_{s}]$, $\kappa =0.03125$. **(b)** $[{V}_{s}]$, $\kappa =0$. **(c)** $[{V}_{s}]$, $\kappa =-\,0.03125$. **(d)** $[{V}_{p}]$, $\kappa =0.03125$. **(e)** $[{V}_{p}]$, $\kappa =0$. **(f)** $[{V}_{p}]$, $\kappa =-\,0.03125$.

**Figure 4:**
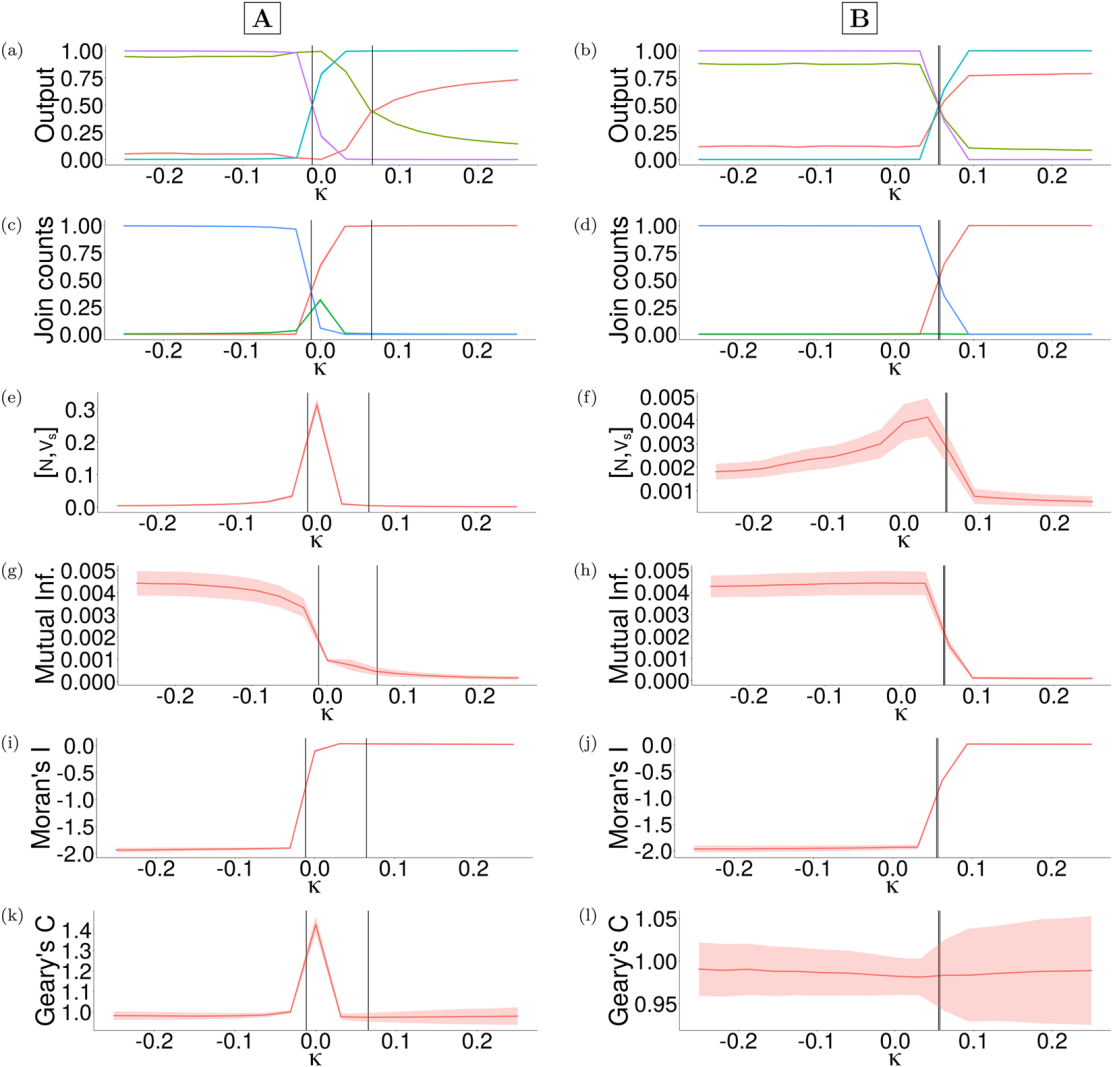
Trends of the EWS’ equilibrium values approaching the transitions of the social and physical dynamics ${K}_{s}$ and ${K}_{p}$ (marked in order by the first and second vertical black lines) respectively, demonstrating the signals given by each tool with respect to the perceived vaccine cost $\kappa$. The intervals in each panel represent one standard deviation of the mean equilibrium value in each stochastic realisation of the model. Social norm $\sigma =0$ for column **(A)**, and $\sigma =0.25$ for column **(B**). **(a,b)** Social dynamics $\langle {V}_{s}\rangle$ (green, solid), $\langle N\rangle$ (red, solid) and physical dynamics $\langle R\rangle$ (black, dashed), $\langle {V}_{p}\rangle$ (blue, dashed). **(c,d)** Join counts: $\langle N,N\rangle$ (blue), $\langle N,{V}_{s}\rangle$ (red), $\langle {V}_{s},{V}_{s}\rangle$ (green). **(e,f)** Dissimilar join count $\langle N,{V}_{s}\rangle$ alone (red). **(g,h)** Mutual information $\langle {\mathscr{M}}\rangle$ (red).** (i,j)** Moran’s I $\langle {\mathscr{J}}\rangle$ (red). **(k,l)** Geary’s C $\langle {\mathscr{C}}\rangle$ (red).

## Results

Due to the low initial vaccine coverage $\alpha =0.05$, all realisations demonstrated an initial epidemic spread (defined in Supplementary Information S3) over the first $7$ weeks. After this period, the dynamics settled down to a quasi-equilibrium state characterized by fluctuations around a mean value that is the focus of our study – the following subsections are grouped by major findings of the model. The term *model variables* refer to the outputs $\langle S\rangle$, $\langle I\rangle$, $\langle R\rangle$, $\langle {V}_{p}\rangle$, $\langle N\rangle$ and $\langle {V}_{s}\rangle$.

### Population vaccine immunity status can differ from aggregate vaccine opinion

Because only susceptible individuals are vaccinated and individuals cannot become ‘unvaccinated’ (but may change their opinion about the vaccine over their lifetime), the population-averaged vaccine opinion is not equal to the population-averaged vaccine immunity status, even at the quasi-equilibrium state. With no social pressure ($\sigma =0$), a small increase in vaccine risk $\kappa \to 0.03125$ pushes the system to endemic infection and anti-vaccine consensus (Fig. 3a) despite a high vaccination rate (Fig. 3d). Towards an explanation, if an agent $n$ is newly birthed into this regime, the probability of having an infected neighbour vanishes $(\langle {I}_{n}\rangle \to 0)$, so that

10 $${{\mathbb{P}}}_{n}(N\to {V}_{s})={{\mathbb{P}}}_{n}({V}_{s}\to N)\approx \frac{1}{2},$$

similar to Eq. (1), with the agents’ probability of being vaccinated over their lifetime as

11 $$0.05+0.95\mathop{\sum }\limits_{m\mathrm{=1}}^{80}\frac{1}{2}{\left(1-\frac{1}{2}\right)}^{m-1}\approx \mathrm{1,}$$

under the assumptions that the average agent with anti-vaccine opinion is almost certain to interact with a disagreeing contact. A similar calculation explains the phenomenon of high vaccination rate (Fig. 3e) despite mixed consensus (Fig. 3b) when vaccine risk becomes neutral ($\kappa \to 0$). A vaccine then perceived as beneficial ($\kappa \to -\,0.03125$) intuitively results in a high vaccination rate (Fig. 3f) and pro-vaccine consensus (Fig. 3c); in both these regimes, the disease survives only through case importation. In the absence of social norms and vaccine risk, the population’s aggregate vaccine opinion may not be a good indicator of its vaccine immunity profile (and *vice versa*); Eq. (2) shows that the probability of changing opinion depends only on ${I}_{n}$ when social norm $\sigma =0$. In this region, the pattern of disease spread will be determined by the initial conditions of the physical dynamics; slight changes in vaccine risk $\kappa$ will push the network towards either of the consensuses, with minimal effect on the high vaccination rate (Fig. 3d). This phenomenon is shared by models V1 and V3, as shown in Supplementary Information S5.1.

### EWS trends identify approaching transitions in both social and physical layers

The trends in both model dynamics and the proposed EWS are shown in Fig. 4, with social norm $\sigma =0$ (column **A**) and $\sigma =0.25$ (column **B**). The first vertical black line in all panels of Fig. 4 represents the transition in the social dynamics ${K}_{s}$, defined as the smallest $\kappa$ value at which $\langle {V}_{s}\rangle \approx \langle N\rangle$ (the mean number of pro-vaccine agents equals the number of anti-vaccine agents); the second vertical black line represents the transition in the physical dynamics ${K}_{p}$, similarly defined as the earliest $\kappa$ where $\langle R\rangle \approx \langle {V}_{p}\rangle$. Multiple physical and social transitions were found for some parameter combinations; these trends and the attendant behaviour of the EWS for models V1 and V3 can be seen in Supplementary Information S5.3. We also note that the equilibrium values of EWS and model variables were averaged over $15-20$ realisations of all parameter combinations.

All EWS show recognisable trends preceding both transitions for both social norm values $\sigma =0$ (column **A**) and $\sigma =0.25$ (column **B**); for instance $\langle N,N\rangle$ (Fig. 4c,d), $\langle N,{V}_{s}\rangle$ (Fig. 4e,f), $\langle {\mathscr{J}}\rangle$ (Fig. 4i,j) and $\langle {\mathscr{C}}\rangle$ (Fig. 4k,l) increase sharply preceding ${K}_{s}$ with all but $\langle N,{V}_{s}\rangle$ (Fig. 4e,f) approaching some maximum value preceding ${K}_{p}$, while $\langle {V}_{s},{V}_{s}\rangle$ (Fig. 4c,d) and $\langle {\mathscr{M}}\rangle$ (Fig. 4g,h) sharply decrease and approach some minimum value before ${K}_{s}$ and ${K}_{p}$ respectively. Mutual information $\langle {\mathscr{M}}\rangle$ (Fig. 4g,h) in particular shows clear changes in trend well before the social transition ${K}_{s}$ occurs. Though $\langle N,{V}_{s}\rangle$ shows a similar rising-falling pattern for both $\sigma =0,\, 0.25$, its maximum value with $\sigma =0.25$ (Fig. 4f) is much lower than that for $\sigma =0$ (Fig. 4e). For $\sigma =0.25$, the mean of the Geary’s C $\langle {\mathscr{C}}\rangle$ (Fig. 4l) shows almost no change, though its envelope broadens post-transition; we see this as a failure of the EWS (no forewarning given). Similar observations hold for model V1 (Supplementary Information S5.2), with the failure of the Geary’s C coefficient ${\mathscr{C}}$ shown clearly in Supplementary Figs. S6 and S7. As stated in the Methods section, the pre- and post-transition trends of the EWS do not generally resemble each other; asymmetry of the EWS about ${K}_{s}$ can be seen Fig. 4 and Supplementary Figs. S6 and S7, showing that (in general) less of a warning is given (if any) when the $\kappa$-series is reversed. This is explicitly demonstrated in Supplementary Figs. S15 and S16, where skewness ${\gamma }_{1}$ is used to quantify asymmetry of the trend of each EWS.

**Figure 5:**
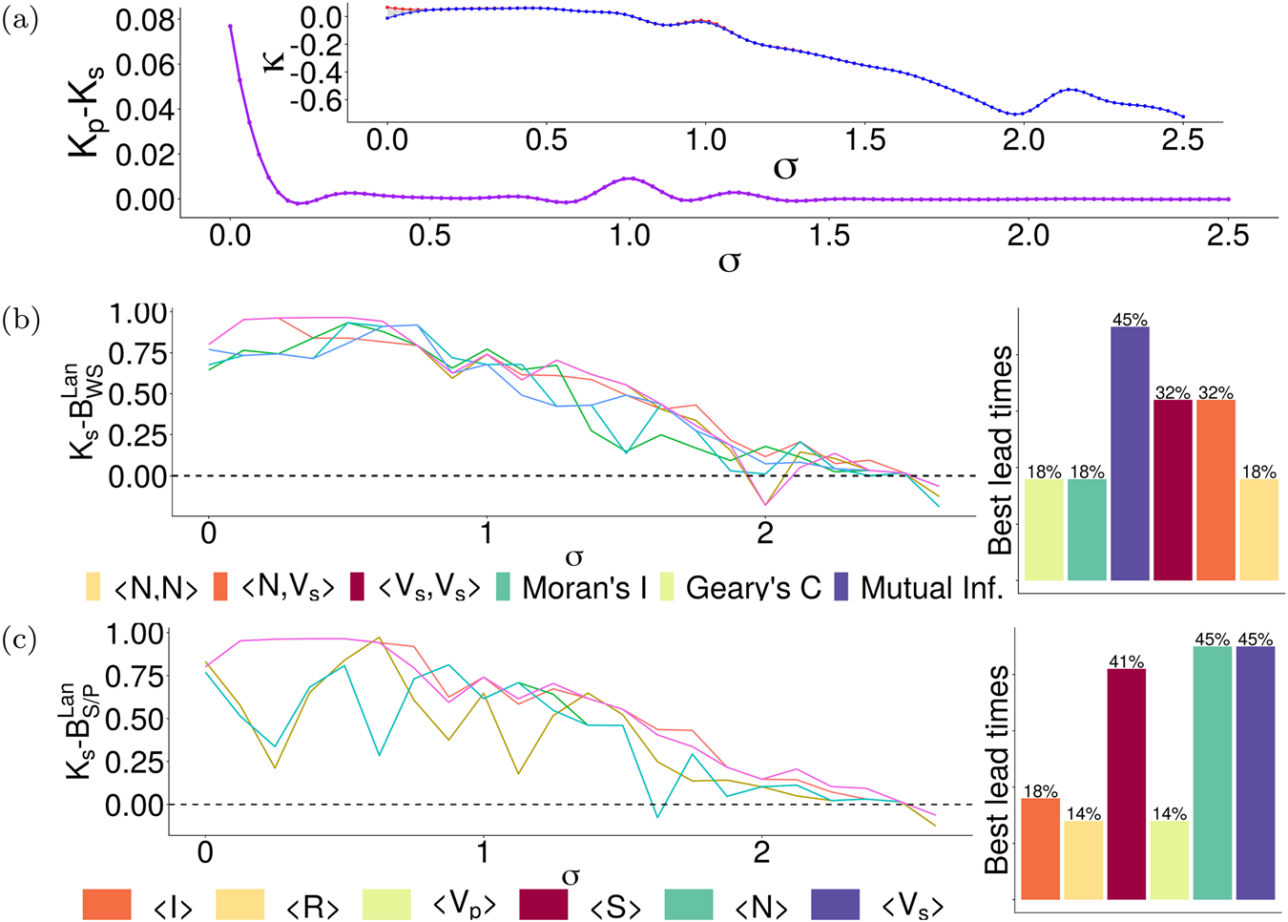
**(a)** Demonstration of the shrinking intertransition distance ${K}_{p}-{K}_{s}$ (purple), with the inset graph showing the estimated locations of ${K}_{s}$ (blue) and ${K}_{p}$ (red). **(b)** Under the Lanzante change point test, the lead time of each EWS $({B}_{{\rm{W}}{\rm{S}}}^{{\rm{L}}{\rm{a}}{\rm{n}}})$ varies substantially with the strength of the social norm $\sigma$; trends corresponding to each EWS are represented by the different colours in the legend; the bar chart on the right gives the number of $\sigma$ values for which each individual EWS gave the maximum lead time. The same for **(c)**, which shows the variance of lead times $({B}_{{\rm{S}}/{\rm{P}}}^{{\rm{L}}{\rm{a}}{\rm{n}}})$ of the model variables with social norm $\sigma$, with the bar chart on the right giving the number of times each model variable gave the maximum lead time of all variables (over all values of sigma).

We can then say that all proposed EWS other than $\langle {\mathscr{C}}\rangle$ give appreciable signals approaching ${K}_{s}$ and ${K}_{p}$ when $\sigma =0.25$ (Fig. 4B). ${K}_{s}$ precedes ${K}_{p}$ (Fig. 4a,b), showing that a shift in consensus will always precede a crisis in vaccination coverage in this model. Also shown is a marked decrease in ${K}_{p}-{K}_{s}$ (the gap between the two transitions ${K}_{s}$ and ${K}_{p}$, which we call the *intertransition distance*) as the social norm strengthens (for example, $\sigma \to 0.25$ in column **(B)** of Fig. 4). The generalisation of these trends to all tested values of $\sigma$ is confirmed in Fig. 5a, where ${K}_{p}-{K}_{s}$ is everywhere positive, though the distance between ${K}_{s}$ and ${K}_{p}$ vanishes with increasing $\sigma$; the inset of Fig. 5a shows the location of ${K}_{s}$ (blue) and ${K}_{p}$ (red) with respect to $\sigma$, so that ${K}_{p}-{K}_{s}$ (purple) gives the width of the area between the two curves in the inset graph at each $\sigma$. Other disparate models of the disease display largely similar concave decreases in the intertransition distance, suggesting that this behaviour arises generally from the model dynamics rather than in some specific subspace of the parameter space (see Supplementary Information S5.2).

### Stronger social norms result in decreased lead time for all EWS

The findings of the preceding subsection are intuitive, as vaccination depends more heavily on individual vaccination opinion than the number of infected neighbouring agents in Eq. (2), so that the opinion dynamics exert more influence than any feedback effect occurring in the physical dynamics. However, the vanishing intertransition distance ${K}_{p}-{K}_{s}$ presents a problem if we depend on predictions of ${K}_{s}$ to enact interventions avoiding the collapse of the system to a non-vaccinated regime (i.e. avoiding ${K}_{p}$). For social norms of increasing strength, we can therefore look at the trend in the *lead time* ${B}_{\ast }={K}_{s}-{\Pi }_{\Psi }$, where ${\Pi }_{\Psi }$ represents some $\kappa$ value at which we can assert that a signal occurs in some sequence $\Psi$ of $\kappa$ values; since we’ve established that ${K}_{s}$ precedes ${K}_{p}$ everywhere, then necessarily any warning of a social transition also warns of the following physical transition, so the quantity ${K}_{p}-{\Pi }_{\Psi }$ is not discussed here.

One way for us to quantify this lead time is to use a *change point detection tool* to find $\kappa$ values at which the two classes EWS (subscript WS) and model variables (subscript S/P) give signals (i.e. display statistically significant changes in trend/behaviour). Here, the Lanzante test^62^ from the trend^63^ package in R is applied to various sequences of equilibrium $\kappa$ values to find change points of EWS (${B}_{{\rm{W}}{\rm{S}}}^{{\rm{L}}{\rm{a}}{\rm{n}}}$, Fig. 5b) and model variables (${B}_{{\rm{S}}/{\rm{P}}}^{{\rm{L}}{\rm{a}}{\rm{n}}}$, Fig. 5c) respectively. (In other words, we computed the change test on the mean of all stochastic realisations at each parameter value combination, rather than computing the change test on each individual time series and then taking the average.) Further discussion of the method of application of this and other change point detection tests to series of $\kappa$ values can be found in Supplementary Information S5.2. *Failure* of a warning signal or model variable $\Psi$ occurs when the warning given comes *after* the social transition, so that ${K}_{s} < {B}_{\Psi }^{{\rm{Lan}}}$.

Looking at the trend in the lead times ${B}_{{\rm{W}}{\rm{S}}}^{{\rm{L}}{\rm{a}}{\rm{n}}}$ (Fig. 5b), the positivity of some curves shows that some of the proposed EWS do indeed give early warnings of coming transitions (largely for $\sigma \le 1.875$). Failures of $\langle N,{V}_{s}\rangle$ and $\langle {\mathscr{M}}\rangle$ occur in the range $1.875\le \sigma \le 2.125$, while all other tests give valid warning signals everywhere $\sigma  < 2.5$; model variables $\langle R\rangle$ and $\langle {V}_{p}\rangle$ fail in the range $1.65\le \sigma \le 2.125$ (Fig. 5c). The failure of all the tests after $\sigma =2.5$ likely results from insufficient length of the EWS’ $\kappa$-series; the inset of Fig. 5a shows that ${K}_{s}\to -\,1$ as $\sigma$ increases. Figure 5c is largely similar, showing failure of all the signals around $\sigma =2.5$ (as in Fig. 5b). In line with our focus on social dynamics as a predictor, $\langle {V}_{s}\rangle$ appears to be the best performing signal of all the model variables; as was reasonably expected, $\langle I\rangle$ appears to perform badly, since its role as a transitory compartment in the disease dynamics means that it never “gathers” sufficient agents over the course of each realisation to give a true indication of the state of the system (other than indicating the presence or absence of endemic infection).

Since the perceived cost of vaccination $\kappa \ge -\,1$ in this study, our method of detecting the change point will not accurately predict a change point ${\Pi }_{\Psi }$ close to $-1$. Since no one warning signal gives the highest lead time for any large contiguous range of $\sigma$ values, there is unfortunately no single objective way to choose a “strongest” signal; they are all suitable tools to predict coming crises in aggregate opinion and vaccination dynamics. However, it is worth noting that mutual information $\langle {\mathscr{M}}\rangle$ and the dissimilar join count $\langle N,{V}_{s}\rangle$ perform better than the other indicators; Fig. 5b shows that mutual information $\langle {\mathscr{M}}\rangle$ gave the largest lead time measured for $\mathrm{45 \% }$ of tested social norm $\sigma$ values, and $\langle {V}_{s},{V}_{s}\rangle$ and $\langle N,{V}_{s}\rangle$ both give the largest lead time for $\mathrm{32 \% }$ $\sigma$ values (multiple EWS showed an identical lead time for some values of $\sigma$). Lead times of all EWS for all three models under other various change point tests are discussed in Supplementary Information S5.4.

### EWS can provide better forewarning than trends in model variables

One final question is whether the proposed EWS (mutual information, Moran’s I, Geary’s C, join counts) give earlier warnings than simply monitoring trends in model variables (such as the number of infections or pro-vaccine agents, using a change point test for prediction in both cases). There are many ways to quantify this, including maximin comparison (finding the larger of minimum values of classes WS and S/P) and maximax comparison (finding the larger of the maxima of each class) at each value of the social norm $\sigma$.

To compare the minima of the EWS and model variable lead times, we define ${\chi }_{{\rm{\min }}}^{{\rm{Lan}}}$ as

12 $${\chi }_{min}^{{\rm{L}}{\rm{a}}{\rm{n}}}=\,min({B}_{{\rm{W}}{\rm{S}}}^{{\rm{L}}{\rm{a}}{\rm{n}}})-\,min({B}_{{\rm{S}}/{\rm{P}}}^{{\rm{L}}{\rm{a}}{\rm{n}}}),$$

and we specify a tolerance ${\varepsilon }_{{\rm{\min }}}^{{\rm{Lan}}}$ to be $\mathrm{1 \% }$ of the total range of ${\chi }_{{\rm{\min }}}^{{\rm{Lan}}}$

13 $${\varepsilon }_{min}^{{\rm{L}}{\rm{a}}{\rm{n}}}=|\frac{max({\chi }_{min}^{{\rm{L}}{\rm{a}}{\rm{n}}})-\,min({\chi }_{min}^{{\rm{L}}{\rm{a}}{\rm{n}}})}{100}|\,.$$

Hence, if ${\chi }_{min}^{{\rm{L}}{\rm{a}}{\rm{n}}} > {\varepsilon }_{min}^{{\rm{L}}{\rm{a}}{\rm{n}}}$, then the EWS is outperforming simple monitoring of trends (model variables).

**Figure 6:**
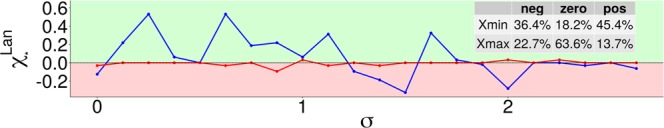
Graph of the trends of ${\chi }_{{\rm{\min }}}^{{\rm{Lan}}}$ (blue) and ${\chi }_{{\rm{\max }}}^{{\rm{Lan}}}$ (red) with respect to the value of the social norm $\sigma$, allowing us to do maximin and maximax comparisons of the two classes of warning signals (WS and model variables S/P). The green-shaded region shows where ${\chi }_{\ast }^{{\rm{Lan}}} > 0$, and the red-shaded region shows where ${\chi }_{\ast }^{{\rm{Lan}}} < 0$. The inset table shows the percentage of $\sigma$ values for which ${\chi }_{\ast }^{{\rm{Lan}}} > {\varepsilon }_{\ast }^{{\rm{Lan}}}$ (*pos*: tracking EWS works better), $|{\chi }_{\ast }^{{\rm{L}}{\rm{a}}{\rm{n}}}|\le {\varepsilon }_{\ast }^{{\rm{L}}{\rm{a}}{\rm{n}}}$ (*zero*: both approaches work equally well) and ${\chi }_{\ast }^{{\rm{Lan}}} < -{\varepsilon }_{\ast }^{{\rm{Lan}}}$ (*neg*: monitoring simple trends works better). **(blue curve, row 1 of inset table)** Positive values (green-shaded region) of ${\chi }_{{\rm{\min }}}^{{\rm{Lan}}}$ occur at the $\sigma$ (social norm) values where the worst-performing (least lead time) EWS still gives higher lead time than the worst-performing model variable. **(red curve, row 2 of inset table)** Similar to above, positive values of ${\chi }_{{\rm{\max }}}^{{\rm{Lan}}}$ occur (in the red-shaded region) when the EWS perform absolutely better than the model variables.

The blue curve in Fig. 6 compares the minima of the EWS (WS) and model variable (S/P) classes (maximin comparison), showing the $\sigma$ values for which the worst-performing (least lead time) EWS $min({B}_{{\rm{W}}{\rm{S}}}^{{\rm{L}}{\rm{a}}{\rm{n}}})$ is either better, *equal* or worse than the worst-performing model variable $min({B}_{{\rm{S}}/{\rm{P}}}^{{\rm{L}}{\rm{a}}{\rm{n}}})$. Points in the green-shaded region represent $\sigma$ values where the EWS’ performance is at worst still better than that of the model variables.

The EWS outperformed simple monitoring of trends in variables for $\mathrm{45.4 \% }$ of the tested $\sigma$ values; maximin comparison shows that EWS are at worst still better than model variables for a large number of $\sigma$ values, with the two classes performing equally badly in $\mathrm{18.2 \% }$ of the $\sigma$ values. Performance of the EWS and the model variables in this test was considered comparable or *equal* if the difference between the two minimum lead times fell under the tolerance ${\varepsilon }_{m}$, so that $|{\chi }_{min}^{{\rm{L}}{\rm{a}}{\rm{n}}}|\le {\varepsilon }_{min}^{{\rm{L}}{\rm{a}}{\rm{n}}}$; performance was equal for $\mathrm{18.2 \% }$ of tested $\sigma$ values, showing that the added computation of the EWS does not always yield a benefit. Otherwise, the points and portion of the blue curve falling in the red-shaded region of Fig. 6 represents values of $\sigma$ where the model variables outperformed the EWS (that is, the minimum lead time of the model variables exceeded the minimum lead time of the EWS); this occurred for $\mathrm{36.4 \% }$ of tested $\sigma$ values.

The second part of the comparison (Fig. 6, red curve) is between the maxima of the lead times; as above, we define the comparison variable ${\chi }_{min}^{{\rm{L}}{\rm{a}}{\rm{n}}}$ and tolerance ${\varepsilon }_{{\rm{m}}{\rm{a}}{\rm{x}}}^{{\rm{Lan}}}$ as

14 $${\chi }_{max}^{{\rm{L}}{\rm{a}}{\rm{n}}}=\,max({B}_{{\rm{W}}{\rm{S}}}^{{\rm{L}}{\rm{a}}{\rm{n}}})-\,max({B}_{{\rm{S}}/{\rm{P}}}^{{\rm{L}}{\rm{a}}{\rm{n}}})\,,\,{\varepsilon }_{max}^{{\rm{L}}{\rm{a}}{\rm{n}}}=|\frac{max({\chi }_{max}^{{\rm{L}}{\rm{a}}{\rm{n}}})-\,min({\chi }_{max}^{{\rm{L}}{\rm{a}}{\rm{n}}})}{100}|\,.$$

The green-shaded portion of Fig. 6 also shows the $\sigma$ values where the EWS outperformed the model variables here, in that the maximum lead time given by the EWS exceed that given by the model variables $({\chi }_{max}^{{\rm{L}}{\rm{a}}{\rm{n}}} > {\varepsilon }_{max}^{{\rm{L}}{\rm{a}}{\rm{n}}})$; points falling within the red-shaded area of Fig. 6 show for which $\sigma$ values the model variables outperform the EWS. From the second row of the inset table in Fig. 6, the two maxima are considered equal $(|{\chi }_{max}^{{\rm{L}}{\rm{a}}{\rm{n}}}|\le {\varepsilon }_{max}^{{\rm{L}}{\rm{a}}{\rm{n}}})$ for $\mathrm{63.6 \% }$ of tested $\sigma$ values, while the EWS outperformed the model variables $({\chi }_{max}^{{\rm{L}}{\rm{a}}{\rm{n}}} > {\varepsilon }_{max}^{{\rm{L}}{\rm{a}}{\rm{n}}})$ for only $\mathrm{13.7 \% }$ of $\sigma$ values.

This shows that the EWS’ lead times are at least equal to those of the model variables for around $\mathrm{63.6 \% }$ of $\sigma$ values and are absolutely larger for $\mathrm{77.3 \% }$ of $\sigma$ values, demonstrating that though monitoring the model variables (both social and physical) is itself valuable, the EWS offer better performance (using the Lanzante change point test). In both (blue and red) curves of Fig. 6, there is no apparent pattern to the positivity/negativity of ${\chi }_{{\rm{\min }}}^{{\rm{Lan}}}$ and ${\chi }_{{\rm{\max }}}^{{\rm{Lan}}}$. These comparisons are given for other tests and models in Supplementary Information S4.

## Discussion

Here we studied a range of early warning signals for critical transitions in a two-layer coupled behaviour-disease model for paediatric infectious diseases. We compared the indicators to one another and the approach of simply monitoring trends in model variables. We found that the performance of the indicators was variable depending on model parameters, but the mutual information statistic and the dissimilar join count showed consistently high pre-transition lead times over various strengths of the social norm, many times giving the highest lead times of all the EWS. Through maximin and maximax comparisons, we found that using EWS provide more advance warning than simply monitoring trends in model variables in a clear majority of cases.

We note that join counts have the additional advantage of easy computability, since they require only counting pairs of a given type. This contrasts with other more computationally intensive indicators such as autocorrelation which require making decisions about whether to study lag-1 or higher order lags, as well as choosing parameter values governing computation of residuals. Moran’s I was also shown to predict the approach of transitions, although perhaps this finding is trivial considering that it is a linear combination of similar and dissimilar join counts. Its predictive power was not as strong as many of the other indicators such as join count and mutual information, hence the added complexity of its calculation may not justify its use. Potential downfalls of the mutual information statistic include its computational complexity and the availability of a suitable data set pairing the personal health of each agent with their social activity.

We also showed that a population may have relatively high vaccine coverage despite a low pro-vaccine opinion. This discrepancy between social and physical dynamics is due to the paediatric infectious disease natural history we assumed in the model. Unlike influenza, where revaccination must occur seasonally, an individual who receives a sufficient number of measles or chickenpox vaccine doses generally has lifelong immunity and therefore the opinion towards the vaccine can decline well before the level of vaccine immunity does. (Individuals can change their opinion but never become ‘unvaccinated’.) The implication of this asymmetry is that monitoring social media networks for changes in opinion using early warning signals like mutual information might provide advance warning of outbreak hot-spots.

The distance between the change point in the EWS indicators and the critical transition in the social dynamics decreases as the social norm grows stronger, as does the distance between the transitions in social and physical dynamics of the model. Given the relative scale of the social norm and vaccine risk parameter values used, stronger social norms decrease the time interval between birth and vaccination decision (the vaccination rate converges to its equilibrium value in fewer time steps than in other regimes); feedback between this and the disease incidence in the network (which affects the number of infected neighbours in each agent’s neighbourhood) alters the probability function controlling the vaccination decisions, effecting faster alignment of majority opinion and vaccination coverage.

This study only lays the foundation for the investigation of spatial EWS for such coupled-behaviour systems. There is much work to be done before they can be meaningfully applied to empirical data. For instance, we assumed that the network was static. This simplifying assumption could be relaxed in future work by using an evolving social dynamic in which agents are allowed to form or break links with new agents based on their node degree^64^ or vaccine opinion and associated social pressures^65^. The rate of interaction between agents was also assumed fixed in our model (relative to the speed of other dynamics, such as the birth/death interval and the length of illness). A valid extension of the model would be a variable rate of communication between agents, since the rate of communication has been shown to influence the rate and efficiency of opinion formation^66^. A further avenue of research would explore interventions to turn populations away from critical transitions. This could answer research questions such as: How far in advance must we act to prevent a social or physical critical transition, and does this change our interpretation of the EWS? If we assume that any of the EWS can be used for monitoring, how would this change in trend alter the reliability of the EWS?

Many researchers and public health bodies are drawing attention to global resurgences of vaccine-preventable illness, and speak to the vast efforts and multiple approaches taken to mitigating outbreaks. A few of these approaches have focused on human behaviour and opinion dynamics, either by directly tracking aggregate vaccine opinion, or monitoring alerts and media reports. Our work demonstrates the potential uses of early warning systems of critical transitions in preventative epidemiology. In particular, our work provides proof-of-concept for the idea of monitoring social networks for early warning signals of both social and epidemiological shifts, and also suggests several EWS indicators that might work well for this purpose.

## Supplementary information

Supplementary information.

## Data Availability

The datasets generated during and analysed during the current study are available from the corresponding author upon reasonable request.
