# Supplementary material for: Spatial early warning signals of social and epidemiological tipping points in a coupled behaviour-disease network
Source: Sci Rep. 2020 May 6;10:7611. doi: 10.1038/s41598-020-63849-0 (PMC7203335; doi:10.1038/s41598-020-63849-0)
Supplement: Supplementary file 1 — Supplementary information. [file 41598_2020_63849_MOESM1_ESM.pdf]

# Spatial early warning signals of social and epidemiological tipping points in a coupled behaviour-disease network

**Brendon Phillips**[1,*], **Madhur Anand**[2], **and Chris T. Bauch**[1]

[1]University of Waterloo, Department of Mathematics, Waterloo, N2L 3G1, Canada
[2]University of Guelph, School of Environmental Sciences, Guelph, N1G 2W1, Canada
[*]b2philli@uwaterloo.ca

# Supplementary Information

## S1 List of Variables

Supplementary Table S1 lists the variables and notation used in the Methods and Results sections.

| State | Description |
|---|---|
| $S$ | susceptible to infection |
| $I$ | infected and infectious |
| $R$ | recovered from illness, immune until death |
| $V_p$ | vaccinated, immune until death |
| $N$ | has an anti-vaccine stance |
| $V_s$ | has a pro-vaccine opinion |
| **Notation** | **Description** |
| $I_n$ | number of infected neighbours |
| $\mathbb{P}_n(\lambda \to \rho)$ | probability of switching sentiment from $\lambda$ to $\rho$, given in equation (1) |
| $Q_n$ | number of neighbours of $n$ |
| $Q_n^\lambda$ | number of neighbours of $n$ with sentiment $\lambda$ |
| $\Delta U_n^{\lambda \to \rho}$ | utility of switching from sentiment $\lambda$ to $\rho$, given in equation (2) |
| $[\lambda]$ | proportion of agents with vaccine opinion $\lambda$ at some time step $\tau$ |
| $\langle \lambda \rangle$ | mean value of $[\lambda]$ over all realisations |
| $[\lambda, \rho]$ | number of edges connecting an agent with vaccine opinion $\lambda$ to another agent with vaccine opinion $\rho$ |
| $\langle \lambda, \rho \rangle$ | mean value of $[\lambda, \rho]$ over all realisations |

**Table S1.** All the variables appearing in the main article.

Since the models used in this paper feature undirected networks, $[\lambda, \rho] = [\rho, \lambda]$ always for sentiments $\lambda$ and $\rho$.

## S2 Flowchart of the model dynamics

Supplementary Fig. S1 gives a flowchart demonstrating the flow of agents through the different phases of the model occurring every time step (for most of the processes). Processes in yellow boxes occur only one throughout the realisation while processes in red boxes denote loops, where the instruction is repeated for all agents in the network. Blue boxes represent true/false decisions, and grey boxes represent choices. The steps are:

1. The realisation is initialised with a ratio $\alpha$ of the agents assigned social state $V_s$ and physical state $V_p$ (i.e., vaccinated pro-vaccine agents), and the remainder assigned the physical state $S$ and social state $N$ (i.e., susceptible anti-vaccine agents). Both layers of the network are given identical random network structure, so that each agent's social contacts are also their physical contacts and *vice versa*.

2. A single susceptible agent is chosen as the index patient of the disease, and infected ($S \to I$).

3. Every susceptible agent in the network interacts with *all* of its physical neighbours, and every such contact carries probability $p$ of infection spread. Since the agent $n$ has $I_n$ many physical contacts, $n$'s total probability of infection in a single time step will be $1-(1-p)^{I_n}$.

4. Every time step, each agent $n$ in the network compares opinion with some random social neighbour $a$, with opinion change only if they disagree (different social states). If they do disagree, then agent $n$ will adopt $a$'s vaccine opinion with probability $P_n$, and is immediately vaccinated if susceptible *and* changing to a pro-vaccine opinion. The next agent in the network is selected, and the loop is repeated until there are no more agents to be considered.

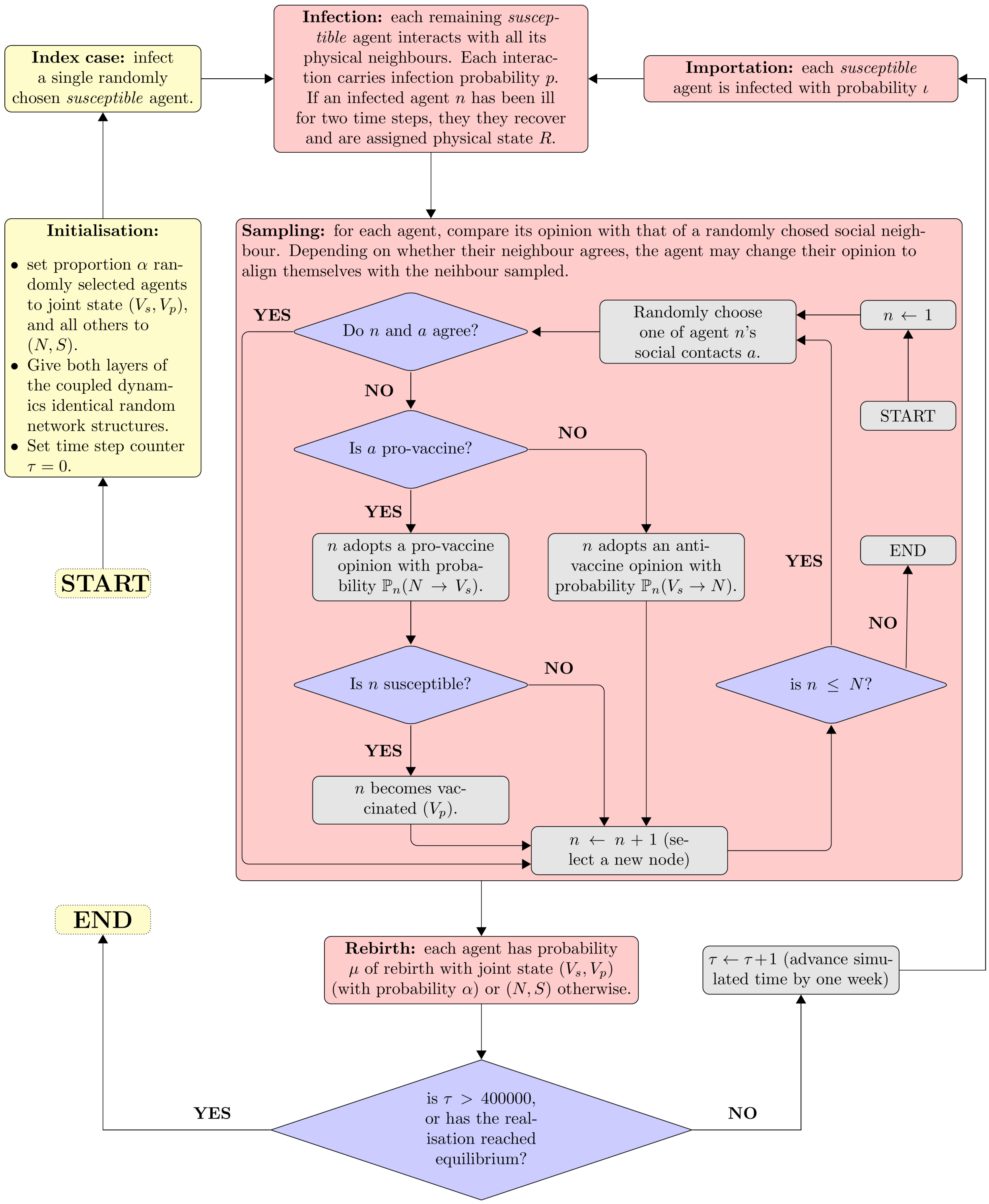


**Figure S1.** Implementation of the model dynamics used for each stochastic realisation. Red blocks represent loops run for each node in the network per time step. Yellow blocks are executed only once per realisation. Blue diamonds represent binary decisions, and grey boxes represent simple instructions.

5. In each time step, every agent faces probability *mu* of death, at which they are instantly replaced by either a vaccinated pro-vaccine agent (joint state $(V_s, V_p)$) with probability $\alpha$, or a susceptible anti-vaccine agent $(N, S)$.

6. Then if the realisation has converged, it ends and the state of the system is output for each time step run; else, it progresses to the next time step.

7. The first process of every subsequent time step is case importation, where proportion $\iota$ of susceptible agents is infected before the infection process is repeated in the simulation.

## S3 Defining 'epidemic' and 'convergence'

We use an agent-based simulation to model heterogeneous mixing among individuals with different opinions and states of health, as well as to impose a random (Erdős-Rényi) network structure on each of the disease and communication networks; not only does this *bottom-up* modelling approach allow the capture of elusive analytically intractable dynamics not captured by differential equation models[1], but also allows us to view the individual trajectory of each agent[2] and track the movement of agents and interactions in dynamic models[3].

Despite arguable necessity, many studies present no explanation of the word "epidemic" when used[4]; here we can define an *epidemic* as a large infection spreading process distinct in size from any established endemic infection rate[5–8], or as a bifurcation ending in an endemic regime[4], both confined to a well-defined region being investigated. For our purposes, the characteristics of an epidemic will be as follows:

1. the transmission and or acquisition of a common (strain of the) disease occurs through effective agent-to-agent contacts or some other common vector[6,8],

2. the time taken for a sudden wave of infection to weaken is small relative to the length of time needed to establish either endemicity or absence of the same disease in the region specified[4],

3. the existence of thresholds in the dynamic for either network and compartment size[9–13], household reproduction number[14], transmissibility[15], average neighbourhood size[16] or behavioural changes[17–19]

4. the spread of the disease is limited only by the number of susceptible agents on the network; given that the transmissibility of the disease exceeds some threshold value, the infection dies only due to the (near-complete) depletion of the pool of susceptible agents (rather than lack of transmission)[5,6,20],

5. the peak number of agents infected during the spread is on the order of the size of the population under study[14],

6. the spreading rate of the infection is superlinear (due to the positive feedback loops formed by interactions between infected and susceptible agents)[5,21,22]. Alternately, the velocity of the epidemic can be measured by established methods given in existing literature[23–25].

A more rigorous treatment of epidemics (and their definition) is given by various sources[4,5].

We specify that any realisation of parameter values has reached a *(computational) equilibrium* when the values of each of $\langle V_s \rangle$, $\langle N \rangle$, $\langle S \rangle$, $\langle I \rangle$, $\langle R \rangle$, $\langle V_p \rangle$, $\langle N,N \rangle$, $\langle N,V_s \rangle$ and $\langle V_s,V_s \rangle$ over the last 500 time steps all have a standard deviation of 0.05% of their respective maximum values. Our model intrinsically satisfies conditions (1), (2), (4), (5) and (6) of Definition S3.

## S4 $\mathscr{I}$ (Moran's I) and $\mathscr{C}$ (Geary's C) in terms of join counts

We support our previous assertion that the Moran's I ($\mathscr{I}$) and Geary's C ($\mathscr{C}$) coefficients are linear combinations of join counts. For this proof, we assume an undirected network where nodes have two states: pro-vaccine ($V_s$ - score 1), and anti-vaccine ($N$ - score 0). Given this convention (also stated in equation (5)), we have that

$$x_k^n = x_k \ \forall n \in \mathbb{N}, \tag{S.1}$$

where $x_k$ represents the score of the $k$th agent. $\omega$ is the adjacency matrix of the network, so that $\omega_{j,k} = 1$ if agents $j$ and $k$ are social neighbours, and $\omega_{j,k} = 0$ otherwise. Using the expression for $\mathscr{I}$ given in equation (6) as

$$\mathscr{I} = \frac{N}{W} \cdot \frac{\sum_{j,k} \omega_{j,k} \cdot (x_j - \bar{x})(x_k - \bar{x})}{\sum_j (x_j - \bar{x})^2}, \tag{S.2}$$

we can derive the expression in equation (7) by first expanding the numerator of equation (S.2) as

$$\sum_{j,k} \omega_{j,k} \cdot (x_j - \bar{x})(x_k - \bar{x}) = \sum_{j,k} \omega_{j,k} \cdot \left(x_j \cdot x_k - (x_j + x_k) \cdot \bar{x} + \bar{x}^2\right) , \tag{S.3}$$

$$= \sum_{j,k} \omega_{j,k} \cdot x_j \cdot x_k - \sum_{j,k} \omega_{j,k} \cdot (x_j + x_k) \cdot \bar{x} + \sum_{j,k} \omega_{j,k} \cdot \bar{x}^2 , \tag{S.4}$$

$$= \underbrace{\sum_{j,k} \omega_{j,k} \cdot x_j \cdot x_k}_{\textbf{(I)}} - 2 \cdot \bar{x} \cdot \sum_{j,k} \omega_{j,k} \cdot x_k + \sum_{j,k} \omega_{j,k} \cdot \bar{x}^2 , \tag{S.5}$$

**(I)** $x_j \cdot x_k$ is nonzero when both $x_j, x_k \neq 0$, so then $j,k \in V_s$; $\sum_{j,k} \omega_{j,k} \cdot x_j \cdot x_k = 2 \cdot [V_s, V_s]$.

$$= 2 \cdot [V_s, V_s] - 2 \cdot \bar{x} \cdot \underbrace{\sum_{j,k} \omega_{j,k} \cdot x_k}_{\textbf{(II)}} + \sum_{j,k} \omega_{j,k} \cdot \bar{x}^2 , \tag{S.6}$$

**(II)** $\omega_{j,k} x_k$ has value when $k \in V_s$ and agent $j$ is any neighbour, so that $\sum_{j,k} \omega_{j,k} \cdot x_k$ considers the number of neighbours of each vaccinator, and $\sum_{j,k} \omega_{j,k} \cdot x_k = 2 \cdot [V_s, V_s] + [N, V_s]$.

$$= 2 \cdot [V_s, V_s] - 2 \cdot \bar{x} \Big(2 \cdot [V_s, V_s] + [N, V_s]\Big) + \bar{x}^2 \cdot \underbrace{\sum_{j,k} \omega_{j,k} \cdot}_{\textbf{(III)}} , \tag{S.7}$$

**(III)** $\sum_{j,k} \omega_{j,k} \cdot$ counts the number of adjacencies between all agent pairs $j$ and $k$, so that $\sum_{j,k} \omega_{j,k} \cdot = W$, where $W$ gives twice the number of unique undirected edges of the network.

$$= 2 \cdot [V_s, V_s] - 2 \cdot \bar{x} \Big(2 \cdot [V_s, V_s] + [N, V_s]\Big) + W \cdot \bar{x}^2 . \tag{S.8}$$

For the denominator of equation (S.2), we have

$$\sum_j (x_j - \bar{x})^2 = \sum_j x_j^2 - 2x_j \cdot \bar{x} + \bar{x}^2 , \tag{S.9}$$

$$= \sum_j x_j^2 - 2 \sum_j x_j \cdot \bar{x} + \sum_j \bar{x}^2 , \tag{S.10}$$

$$= \sum_j x_j - 2 \cdot \bar{x} \underbrace{\sum_j x_j}_{\textbf{(IV)}} + \bar{x}^2 \underbrace{\sum_j}_{\textbf{(V)}} , \tag{S.11}$$

**(IV)** $x_j$ has value only when $j \in V_s$, so that $\sum_j x_j = [V_s]$ gives the number of pro-vaccine agents.

**(V)** $\sum_j$ sums every node in the network, so that we get the number of nodes in the network; $\sum_j = N$.

$$= [V_s] - 2\bar{x} \cdot [V_s] + N\bar{x}^2 , \tag{S.12}$$

$$= (1 - 2\bar{x}) \cdot [V_s] + N\bar{x}^2 , \tag{S.13}$$

$$= \gamma \cdot [V_s] + N \cdot \bar{x}^2 , \tag{S.14}$$

where

$$\gamma = 1 - 2\bar{x} . \tag{S.15}$$

Therefore, the full expression of Moran's I is written

$$\mathscr{I} = \frac{N}{W} \cdot \frac{2 \cdot [V_s, V_s] - 2\bar{x} \cdot (2 \cdot [V_s, V_s] + [N, V_s]) + W \cdot \bar{x}^2}{\gamma \cdot [V_s] + N \cdot \bar{x}^2}, \tag{S.16}$$

which can be seen as a linear combination of join counts

$$\mathscr{I} = \frac{N}{(\gamma \cdot [V_s] + N \cdot \bar{x}^2) \cdot W} \left( (2 - 4\bar{x}) \cdot [V_s, V_s] - 2\bar{x} \cdot [N, V_s] + W \cdot \bar{x}^2 \right). \tag{S.17}$$

Using the expression for the Geary's C statistic $\mathscr{C}$ given in equation (8) as

$$\mathscr{C} = \frac{N-1}{W} \frac{\overbrace{\sum_{j,k} \omega_{j,k} \cdot (x_j - x_k)^2}^{\textbf{(VI)}}}{\sum_j (x_j - \bar{x})^2}, \tag{S.18}$$

we can identify the numerator **(VI)** with the expression given for $[N, V_s]$ in equation (4) , so that

$$\sum_{j,k} \omega_{j,k} \cdot (x_j - x_k)^2 = 2 \cdot [N, V_s]. \tag{S.19}$$

Since the denominator of **(VI)** is identical to that of equation (S.2), the entire expression for $\mathscr{C}$ can be written as

$$\mathscr{C} = \frac{N-1}{W} \frac{2 \cdot [N, V_s]}{\gamma \cdot [V_s] + N \cdot \bar{x}^2}. \tag{S.20}$$

## S5 Comparisons of models V1, V2 and V3

### S5.1 Model parameters

We use three disparate models V1, V2 and V3 to investigate these EWS, with their respective parameter values and ranges shown in Supp. Table S2. The Results section gives the results for model V2, and here we compare the results of the other models V1 and V3.

| Parameter | Interpretation | V1 | V2 | V3 |
|---|---|---|---|---|
| $N$ | number of agents | 10000 | 40000 | 562500 |
| $\iota$ | case importation (proportion of susceptible agents) | $2.5 \times 10^{-4}$ | $1 \times 10^{-5}$ | $2.5 \times 10^{-5}$ |
| $p$ | disease infectivity | 0.2, 0.8 | 0.2, 0.8 | 0.8 |
| $\xi_*$ | random sentiment change | $\xi_1 = 1 \times 10^{-4}$ | | $1 \times 10^{-4} \leq \xi_{52} \leq 0.1$ |
| $\langle Q_n \rangle$ | mean neighbourhood size | 30 | | 50 |
| $\sigma$ | strength of social norm | $0 \leq \sigma \leq 3$ | | $0 \leq \sigma \leq 6$ |
| $\kappa$ | perceived risk risk of adverse vaccine effects | $-1 \leq \kappa \leq 1$ | | $-2 \leq \kappa \leq 6$ |
| $\mathscr{T}$ | length of each realisation | $10000 \leq \mathscr{T} \leq 40000$ and until equilibrium | | 11000 |
| $\alpha$ | initial pro-vaccine proportion | 0.05, 0.95 | | |
| $\ell$ | duration of illness | 2 weeks (time steps) | | |
| $\mu$ | birth/death rate | $2.4 \times 10^{-4}$ | | |

**Table S2.** A table of the baseline parameter values used for each simulation. $N$ represents the number of agents in the simulation, $\xi_1$ represents the probability of random sentiment switch per time step, and $\xi_{52}$ represents the probability of randomly switching sentiment once per year (52 time steps).

Models V1 and V3 were parametrised in the way outlined in the Results section: $\kappa = 0$, $\sigma = 0$ and $\alpha = 0.05$ give the result $\langle R \rangle < 0.05$ at equilibrium (defined in Supp. Information S3). The uniform birth/death rate $\mu = 2.5 \times 10^{-4}$ was chosen

to give an average life expectancy of 80 years, and a duration of illness $\ell = 2$ was chosen since the length of the infectious period. Different sizes $N$ were chosen to allow for a sensitivity analysis, given that the three models present major differences in infectivity ($p$), mean neighbourhood size ($\langle Q_n \rangle$, hence the number of effective contacts per week) and noise ($\xi_1$ and $\xi_{52}$), as can be seen in Supp. Table S2.

All the transitions shown occur for almost identical values of the social norm $\sigma$, suggesting that variance of the noise parameter $\xi_{52}$ has minimal effect over the dynamics of V3. The dynamics of model V2 was shown to be sensitive to slight change in the perceived vaccine risk $\kappa$ when $\sigma = 0$ (Fig. 3). Models V1 (Supp. Fig. S3) and V3 (Supp. Fig. S2) feature similar instabilities to model V2.

For model V3, a slight increase in $\kappa$ from $\kappa = 0$ (Supp. Fig. S2b, e) to $\kappa = 0.01$ (Supp. Fig. S2a, d) pushes the system to an anti-vaccine consensus when $\xi_{52} = 1 \times 10^{-4}$, while a decrease to $\kappa = -0.01$ (Supp. Fig. S2c, f) gives a shift to the opposite pro-vaccine consensus. The similarity in behaviour (trends in the time series) between Supp. Fig. S2a-f ($\xi_{52} = 1 \times 10^{-4}$), Supp. Figs. S2g-l ($\xi_{52} = 1 \times 10^{-3}$) and S2m-r ($\xi_{52} = 1 \times 10^{-2}$) show that increasing the noise present in the model doesn't affect this sensitivity. Similar observations hold for model V1 for both infectivities $p = 0.2$ (Supp. Fig. S3a-f) and $p = 0.8$ (Supp. Fig. S3g-l), where slight changes to the vaccine risk $\kappa$ result in either anti-vaccine (Supp. Fig. S3a, g) or pro-vaccine (Supp. Fig. S3c, i) consensus. One difference between models V2 and V3 is the phenomena of Supp. Figs. S3d and S3j, where the increase in $p$ from 0.2 to 0.8 has negatively affected the physical vaccination rate; increasing the infection rate from $p = 0.2$ to $p = 0.8$ takes $[V_p]$ from 0.85 (Supp. Fig. S3d) to 0.5 (Supp. Fig. S3j).

We stated in the Results section that the parameter ranges $\kappa \in [-1, 1]$, $\sigma \in [0, 3]$ sufficiently captured transitions in both dynamics as well as the behaviours of the EWS; this is shown for model V2 in Supp. Fig. S4, and for V1 and V3 in Supp. Fig. S5.

### S5.2 Intertransition distance $K_p - K_s$

Models V1 and V3 demonstrate a vanishing intertransition distance similar to model $V2$ in Supp. Fig. S8, where the distance between the two vertical black lines (representing $K_s$ and $K_p$) in Fig. 4a decreases as the social norm gets stronger in Fig. 4b; this shrinking distance between the two vertical lines can also be seen in Supp. Figs. S6 and S7 by comparing columns **(A)** and **(B)**. The independence of these trends in the intertransition distance to the difference in the three models was expected; they follow immediately from previous discussions in which few substantive differences were seen. Since this is then a property of the dynamics themselves, any discussion about the interpretation and validity of the EWS will be largely identical among the three models.

### S5.3 Multiple definable transitions $K_s$ and $K_p$

A difficulty with the interpretation of Fig. 5a (and all panels of Supp. Fig. S8) is the case where there are multiple social ($K_s$) and physical ($K_p$) transitions. The definitions of $K_s$ and $K_p$ given in the Results section rely on finding the *earliest* $\kappa$ value where $\langle V_s \rangle \sim \langle N \rangle$ and $\langle V_p \rangle \sim \langle R \rangle$ respectively. For model V2, this problem occurs in the range $\sigma \geq 1$ (Supp. Fig. S9). Of particular interest are the trends in the proposed EWS; for V2 the behaviour of $\langle \mathscr{M} \rangle$ (mutual information) in this range is shown in column **(B)** of Supp. Fig. S9. There is still a change in trend approaching the transitions $K_s$ and $K_p$ (which occur almost simultaneously), as shown in Fig. 5a ($K_p \sim K_s$ when $\sigma > 1$); since both the warnings from the EWS and the definitions of $K_s$ and $K_p$ depend on the lowest $\kappa$ values, the EWS still give early warning signals despite this.

Supplementary Fig. S9m details the number of transitions in the physical (#$K_p$, red) and social (#$K_s$, blue) dynamics per value of the social norm $\sigma$; the height of each bar represents the total number of transitions for the corresponding value of the social norm $\sigma$, and the length of each coloured portion of the bar represents the number of transitions of that type. For example, there are 23 definable transitions when $\sigma = 1.875$; 15 are social transitions (the length of the red portion of that bar), and the remaining 8 transitions occur in the physical dynamics ($23 - 15 = 8$). For model V2, multiple transition (#$K_* > 1$) occurs in the region $\sigma > 1$. However, our description of a transition also counts occurrences where the two opposing curves merely touch each other (as best as can be determined from an ensemble of stochastic realisations, rather than the result of some rigorous analysis), thereby possibly inflating the value of the number of *meaningful* transitions producing changes in the vaccination rate or aggregate sentiment.

A similar observation can be made with model V1 in the range $\sigma > 0.5$; the trends of the dissimilar join count $\langle N, V_s \rangle$ (column **(B)**, Supp. Fig. S10) still demonstrate statistically significant change before either transition despite the irregularity of the trends shown (as compared to those occurring in the region $\sigma \geq 0.75$). Supplementary Fig. S10i and S10j show a marked increase in the intertransition distance (the gap between the two horizontal lines), also shown by the sudden change in trend shown in Supp. Fig. S8a in the range $2 < \sigma < 2.125$. This represents a region of the parameter space of model V1 where the physical transition $K_p$ lagged the social transition $K_s$ far more than expected due to the occurrence of a social transition *without an accompanying physical transition*. This assertion is supported by Supp. Fig. S8m, where the bar chart detailing the numbers of transitions shows that there are many more social transitions than physical transitions (#$K_s >$ #$K_p$). Previously, even though there were multiple transitions $K_s$ and $K_p$, the first social transition was always a reliable predictor of the first physical transition;

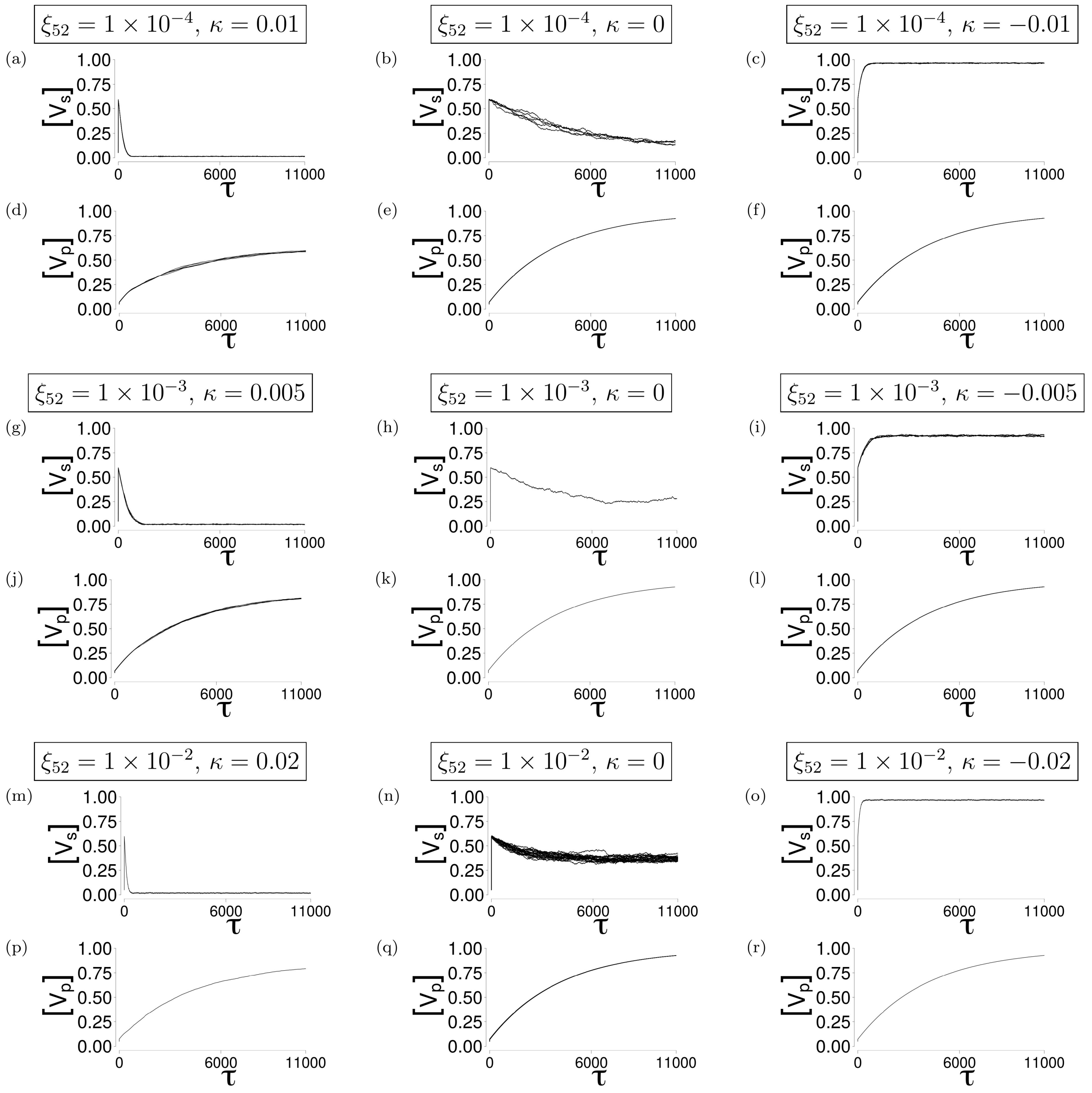


**Figure S2.** In the absence of a social norm ($\sigma = 0$), varying the noise parameter $\xi_{52}$ does not affect the behaviour of model V3 when the perceived vaccine risk $\kappa$ is close to zero (similar to model V2). These time series demonstrate the sensitivity of the social dynamics **(a-c,g-i,m-o)** of model V3 to small changes in $\kappa$ for various values of $\xi_{52}$. $[V_p]$ represents the number of vaccinated agents in each time step, and $[V_s]$ gives the number of pro-vaccine agents. Each panel presents time series from 20 realisations of the parameter combination.

here, there are multiple social transitions before the first physical transition is seen; this presents a different opportunity for the misjudgement of the proximity of a vaccine crisis, since this may lead to a large lead time in parameter regions presenting otherwise smaller lead times. For example, if we expect a vaccine crisis to follow soon after a shift in aggregate sentiment, then a detected social transition may lead to time-sensitive preparations for a vaccine crisis that occurs far later than expected.

In sum, all the values of $K_s$ and $K_p$ estimated in this study may not represent the earliest definable transition in the dynamics, but rather the first definable transition *within the range* $-1 \leq \kappa \leq 1$. As will be shown in Supp. Information S5, different

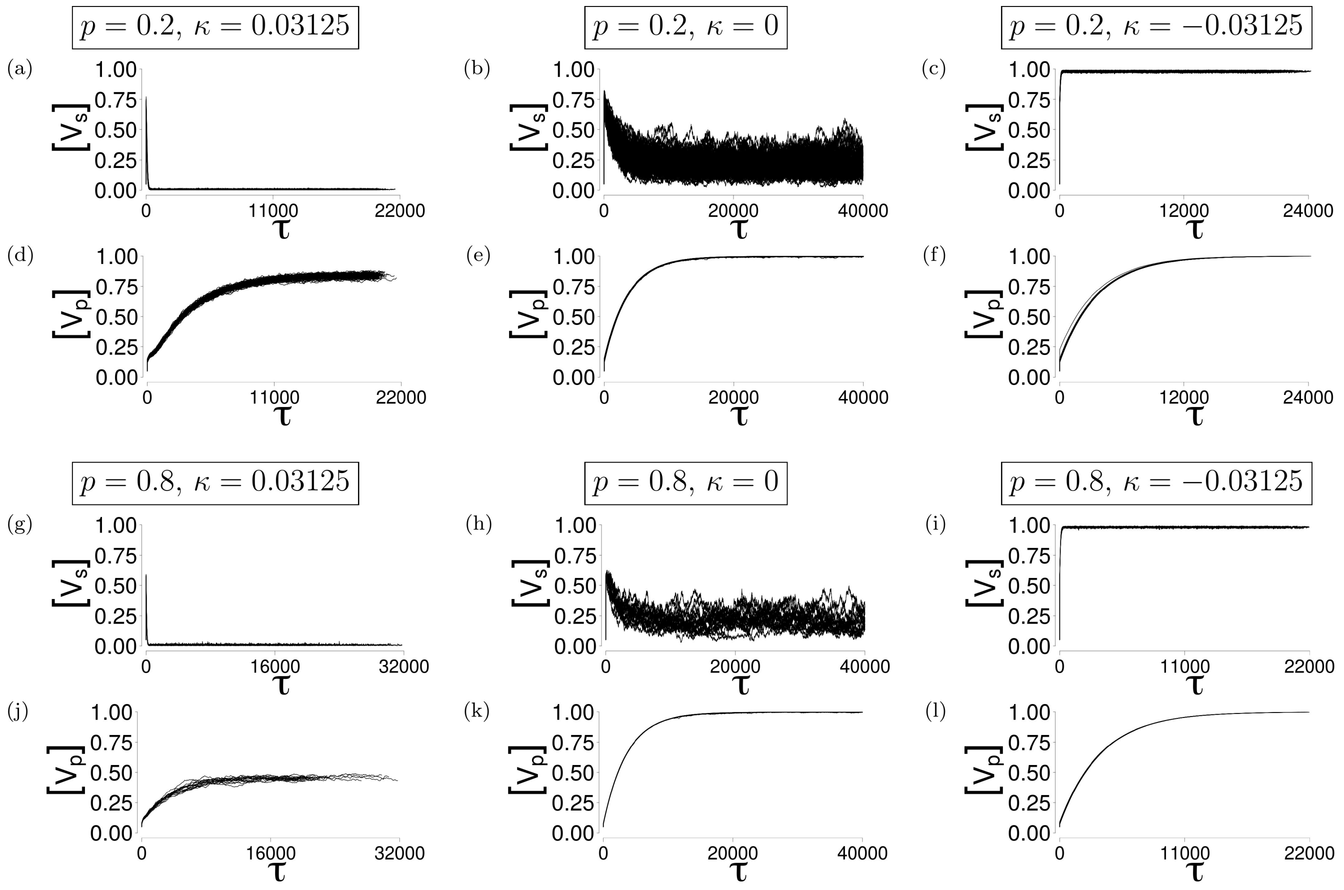

**Figure S3.** In the absence of a social norm ($\sigma = 0$), increasing the infectivity of the disease does not alter the sensitivity of the social dynamics of model V1 (similar to models V2 and V3), but does change the vaccination rate when $\kappa$ becomes positive. $[V_p]$ represents the number of vaccinated agents in each time step, and $[V_s]$ gives the number of pro-vaccine agents. Each panel presents time series from 20 realisations of the parameter combination.

models (specifically different values of $N$) show a wider range of transitions of the values $K_s$ and $K_p$.

## S5.4 Change point testing and warnings of the EWS

Since $15-20$ realisations were run for each unique tuple of parameter values, averaged time series were made by finding the mean of all time series at each time step $t$. For each value of $\sigma$, a corresponding $\kappa$-series was formed by assembling the means of the last 500 time steps of each averaged time series for each value of $\kappa$. Per value of $\sigma$, each change point test was applied to the $\kappa$-series as follows: the series for each EWS was ordered in increasing values of $\kappa$, and the change point test was applied to successively longer segments of the sequence (of length at least 3, starting from the first value $\kappa = -1$) until a predicted change point $B_*$ was returned with a certainty $p < 0.05$. Four change point tests were used: the Buishand test[26], the Pettitt test[27,28], the Lanzante test[29] (used in the main text), and the Standard Normal Homogeneity test[30,31] (hereon referred to as the *SNHT*).

This method can be justified by the various panels of Supp. Fig. S11, which show how the warnings of each EWS depend on the length of the $\kappa$-series of values the test was applied to. For example, the purple line and points in Supp. Fig. S11l show the warning given by the $\langle N,N \rangle$ count under the SNHT test for model V2. Starting (in all instances) from $\langle V_s \rangle_{\kappa=-1}$, applying the SNHT to a series of length 4 gives a statistically insignificant result ($p \approx 0.8$); there is a corresponding purple filled point at $n = 4$ on the $x$-axis, and the same explanation holds for $4 \leq n \leq 7$. For $n = 8$ on the $x$-axis (representing a sequence of length 8), we see the first statistically significant result under the SNHT ($p = 0.02$), represented by the start of the purple line.

As progressively longer sequences of $\langle V_s \rangle_{\kappa \geq -1}$ are used, the SNHT continues to give predictions with $p$ values under the 0.05 threshold of significance (as evidenced by the continuation of the line in Supp. Fig. S11l, rather than the sudden appearance of filled points), though $B^{\text{SNHT}}$ itself is increasing. Interestingly, as $n \to 30$, $B^{\text{SNHT}}$ approaches the value of $K_s$, and the other $B_*$ ($B_*^{\text{BR}}$ in red, $B_*^{\text{Lan}}$ in blue and $B_*^{\text{Pet}}$ in green) also show the same increasing trend in the final moments before the arrival of $K_s$; all panels of Supp. Fig. S11 end at $n = 32$, since this is when the end point of the $\kappa$-series used for each change

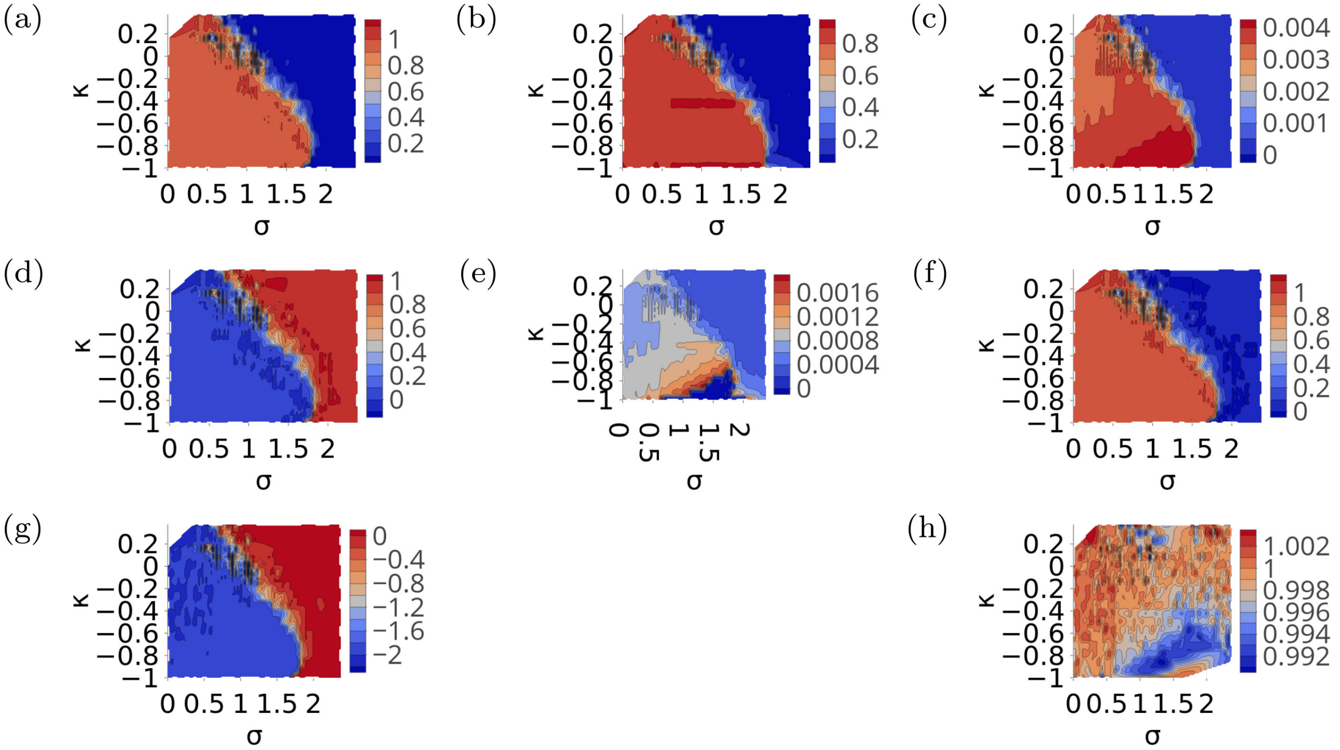


**Figure S4.** Contour plots of the subregion $(\kappa, \sigma) \in [-1, 0.2] \times [0, 2.4]$ of the parameter range of model V2, showing correspondence between the values of $\langle V_s \rangle$ and $\langle V_p \rangle$, and the proposed EWS. **(a)** $\langle V_s \rangle$. **(b)** $\langle V_p \rangle$. **(c)** $\mathscr{M}$. **(d)** $\langle N, N \rangle$ . **(e)** $\langle N, V_s \rangle$ . **(f)** $\langle V_s, V_s \rangle$ . **(g)** $\langle \mathscr{I} \rangle$. **(h)** $\langle \mathscr{C} \rangle$.

point test reaches or passes $K_s$ (there is little use here for predictions of $K_s$ given *after* the transition has occurred).

For model V2, comparing the panels of column **(C)** of Supp. Fig. S11 shows that the $\langle N, N \rangle$ and $\langle N, V_s \rangle$ counts (Supp. Figs. S11l and S11o respectively) give the earliest significant warnings under the SNHT (as seen from the early start of the solid purple lines representing $B^{\mathrm{SNHT}}$). Conversely, the latest warnings come from $\langle V_s, V_s \rangle$ (Supp. Fig. S11r) under all four change point tests, since the earliest significant result occurs at $n = 18$. This seems to be due to the relative value of the join counts; $\langle V_s, V_s \rangle = \mathscr{O}(N^2)$ while $\langle N, N \rangle$ and $\langle N, V_s \rangle$ are both small before $K_s$, so that the establishment of a trend on $\langle N, N \rangle$ and $\langle N, V_s \rangle$ will be "more noticeable" than that for $\langle V_s, V_s \rangle$ .

While the signals of the join counts $\langle N, N \rangle$ , $\langle N, V_s \rangle$ and $\langle V_s, V_s \rangle$ increase under all change point tests as $K_s$ is approached (Supp. Fig. S11j-r), $\langle \mathscr{C} \rangle$, $\langle \mathscr{I} \rangle$ and $\langle \mathscr{M} \rangle$ remain relatively stable as more terms in the $\kappa$-series are added to the test (Supp. Fig. S11a-i). Our interpretation of this difference is neither positive or negative, since a stable prediction inspires trust in the result, whereas the establishment of an increasing trend in the warnings of the join counts seems a better indicator of the proximity of $K_s$ than the estimate given by the test (this is a case where the initial value and trends of the result are both useful). Since this increase in the warnings of $\langle N, N \rangle$ , $\langle N, V_s \rangle$ and $\langle V_s, V_s \rangle$ (Supp. Fig. S11j-r) persists under all the change point tests used, this trend can be seen as a property of the $\kappa$-series of the join counts *and hence the join counts themselves as EWS* rather than a spurious observation. A similar analysis holds for model V1 in columns **(A)** and **(B)** of Supp. Fig. S11, where again $\langle N, N \rangle$ and $\langle N, V_s \rangle$ give the earliest warnings of the approach of $K_s$ for $p = 0.2$ (Supp. Fig. S11j, m) and for $p = 0.8$ (Supp. Fig. S11k, n).

Measurements of the lead time for transitions $K_s$ under the Lanzante test are shown in Figs. 5c-d; here, we show the lead times given by other change point tests used on the EWS for model V2. Comparing the panels of Supp. Figs. S12, S13 and S14, all EWS retain their validity under the various tests, except for the consistent failure of the EWS at higher values of $\sigma$ for both models (V1 and V2) previously attributed to insufficient range of $\kappa$ as $\sigma$ increased. Of all the EWS, the performance of $\langle V_s, V_s \rangle$ and $\langle \mathscr{C} \rangle$ vary the most with respect to the test used for both V1 and V2. For instance, under the SNHT (Supp. Fig. S12g), $\langle \mathscr{C} \rangle$ is the only failure of all the EWS, with a negative lead time in the range $1.75 \leq \sigma \leq 2.5$ (i.e. the warning occurs post-transition). Also of note is that for model V2, the Lanzante test (Supp. Fig. S12c, d) not only gives the highest maximum lead times for all the EWS, but also yields a consistently larger average lead time than the other change point tests. It appears that the Lanzante test provides the largest lead times in the range $\sigma < 2$ for V2.

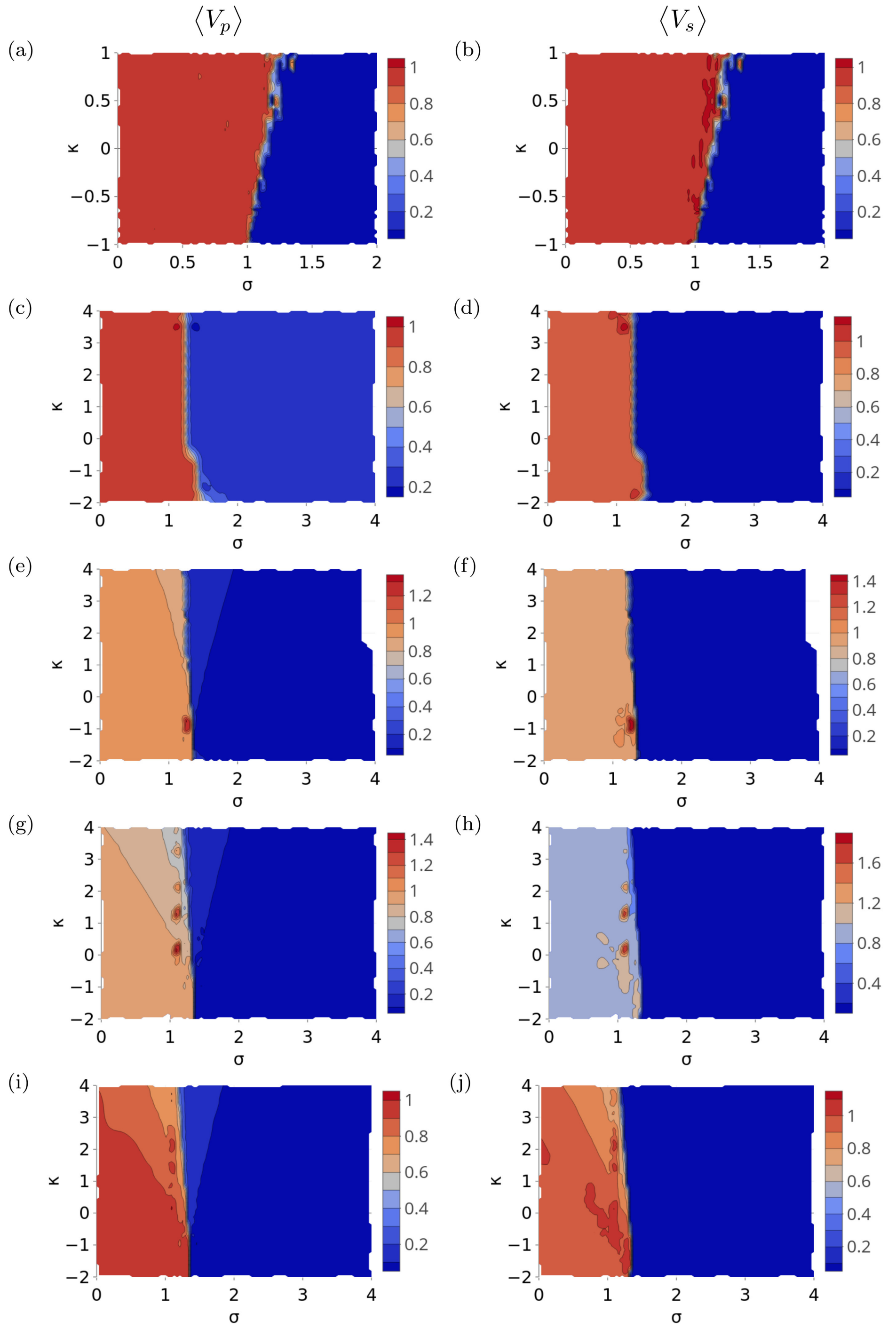


**Figure S5.** Contour plots of $\langle V_p \rangle$ **(Row 1)** and $\langle V_s \rangle$ **(Row 2)** for the models V1 **(a-b)** and V3 **(c-j)** showing that the investigated parameter regions capture the transitions $K_s$ and $K_p$. **(a-b)** model V1, $p = 0.8$. **(c-d)** $\xi_{52} = 0.1$. **(e-f)** $\xi_{52} = 0.01$. **(g-h)** $\xi_{52} = 0.001$. **(i-j)** $\xi_{52} = 0.0001$.

Similar observations cannot be made for model V1, however; important differences in Supp. Figs. S13 and S14 are that $\langle V_s, V_s \rangle$ is also now inconsistent (along with $\langle \mathscr{C} \rangle$), with earlier failure than in model V2 at $\sigma = 1.21875$ as well as $\sigma > 1.90625$ when $p = 0.2$. This can possibly be attributed to behaviours due to the different parameter values of the models, and or the finer resolution of $\sigma$ values in model V1 as compared to model V2; values of $\sigma$ increase in increments of 0.03125 in model V1, whereas the finest increment of $\sigma$ is 0.125 in model V2. Similar to model V2, $\langle \mathscr{I} \rangle$ and $\langle N, V_s \rangle$ perform well under all the change tests with relatively high lead times when $p = 0.2$, but $\langle N, N \rangle$ now also gives a lead time comparable to the two

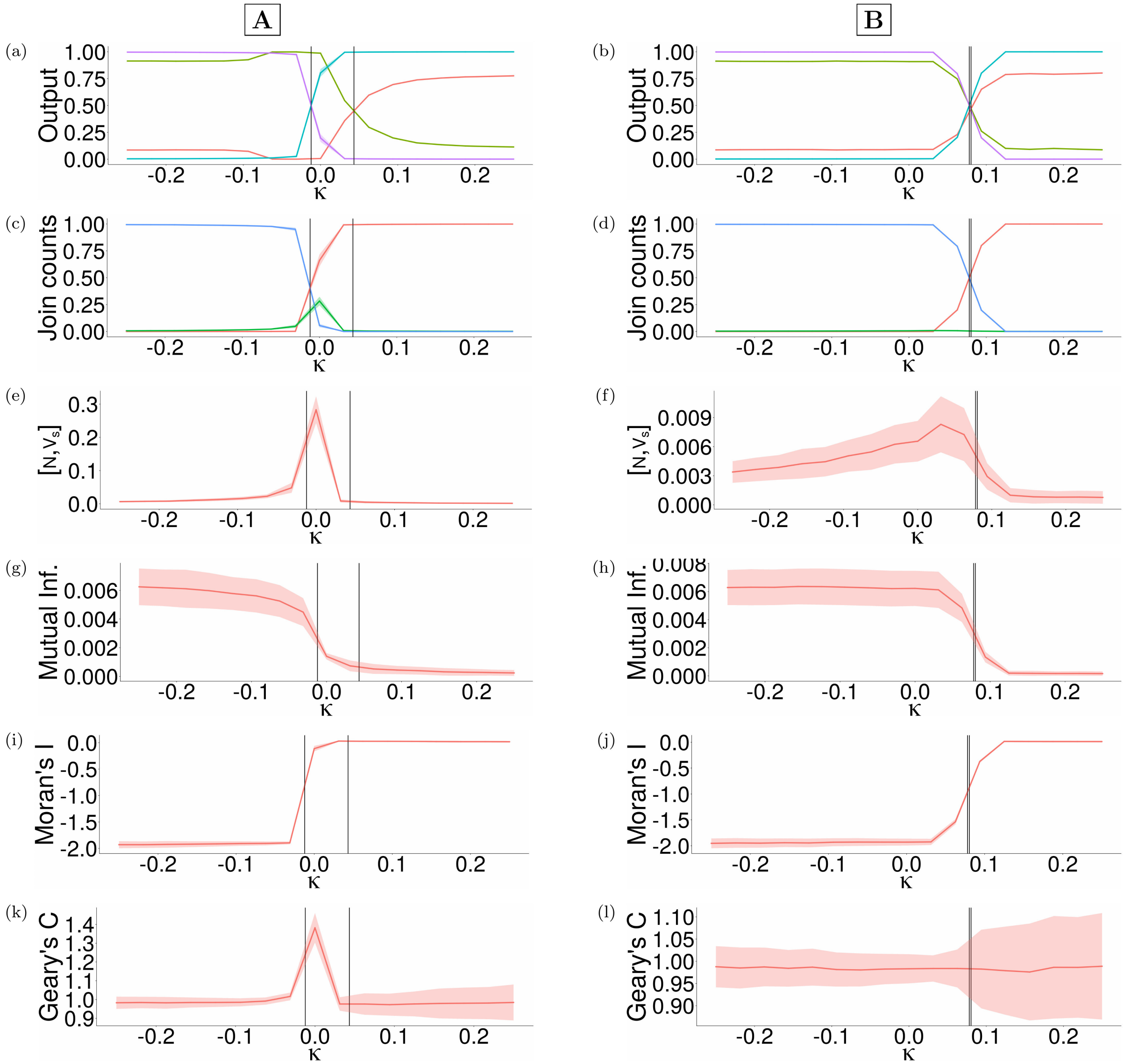


**Figure S6.** Trends of the EWS' equilibrium values in model V1 with infectivity $p = 0.2$ approaching the transitions of the social and physical dynamics $K_s$ and $K_p$ (marked by the first and second black lines) respectively, demonstrating the signals given by each tool with respect to the perceived vaccine cost $\kappa$. The intervals in each panel represent one standard deviation of the mean equilibrium value in each stochastic realisation of the model. Social norm $\sigma = 0$ for **(column A)**, and $\sigma = 0.25$ for **(column B)**. **(a-b)** Social dynamics $\langle V_s \rangle$ (green, solid), $\langle N \rangle$ (red, solid) and physical dynamics $\langle R \rangle$ (black, dashed), $\langle V_p \rangle$ (blue, dashed). **(c-d)** Join counts: $\langle N,N \rangle$ (blue), $\langle N,V_s \rangle$ (red), $\langle V_s,V_s \rangle$ (green). **(e-f)** $\langle N,V_s \rangle$ alone. **(g-h)** Mutual information $\langle \mathscr{M} \rangle$. **(i-j)** Moran's I $\langle \mathscr{I} \rangle$. **(k-l)** Geary's C $\langle \mathscr{C} \rangle$.

previous EWS. Finally, there is not as much variation in maximum lead time per EWS for model V1 as there is for model V2; maximum lead times for the best EWS ($\langle \mathscr{M} \rangle$, $\langle N,V_s \rangle$ and $\langle V_s,V_s \rangle$ ) are all around 1.

### S5.5 Reversibility of the $\kappa$-series of the EWS

Column **(A)** of Supp. Fig. S15 plots the skewness $\gamma_1$ of each trend with respect to the intertransition distance $K_p - K_s$, while column **(B)** plots the skewness $\gamma_1$ against the strength of the social norm $\sigma$; column **(B)** suggests a relationship between the skewness $\gamma_1$ and the social norm $\sigma$, though it may not be causative. As an example of skewness in the case of a weaker social norm $\sigma \approx 0$, trends in the mutual information $\langle \mathscr{M} \rangle$ (Supp. Fig. S15b) and Moran's I $\langle \mathscr{I} \rangle$ (Supp. Fig. S15d) are symmetric (i.e., small $\gamma_1$) for small $\sigma$. As $\sigma$ increases however, the direction of $\kappa$ becomes vital to the calculation and interpretation of warning signals; for example, Supp. Fig. S15j shows a clear change in the skewness of the dissimilar join count $\langle N,V_s \rangle$ as $\sigma \to 0.25$. This change is visible moving between Figs. 4e ($\sigma = 0$) and 4f ($\sigma = 0.25$), where the trend becomes more left-skewed.

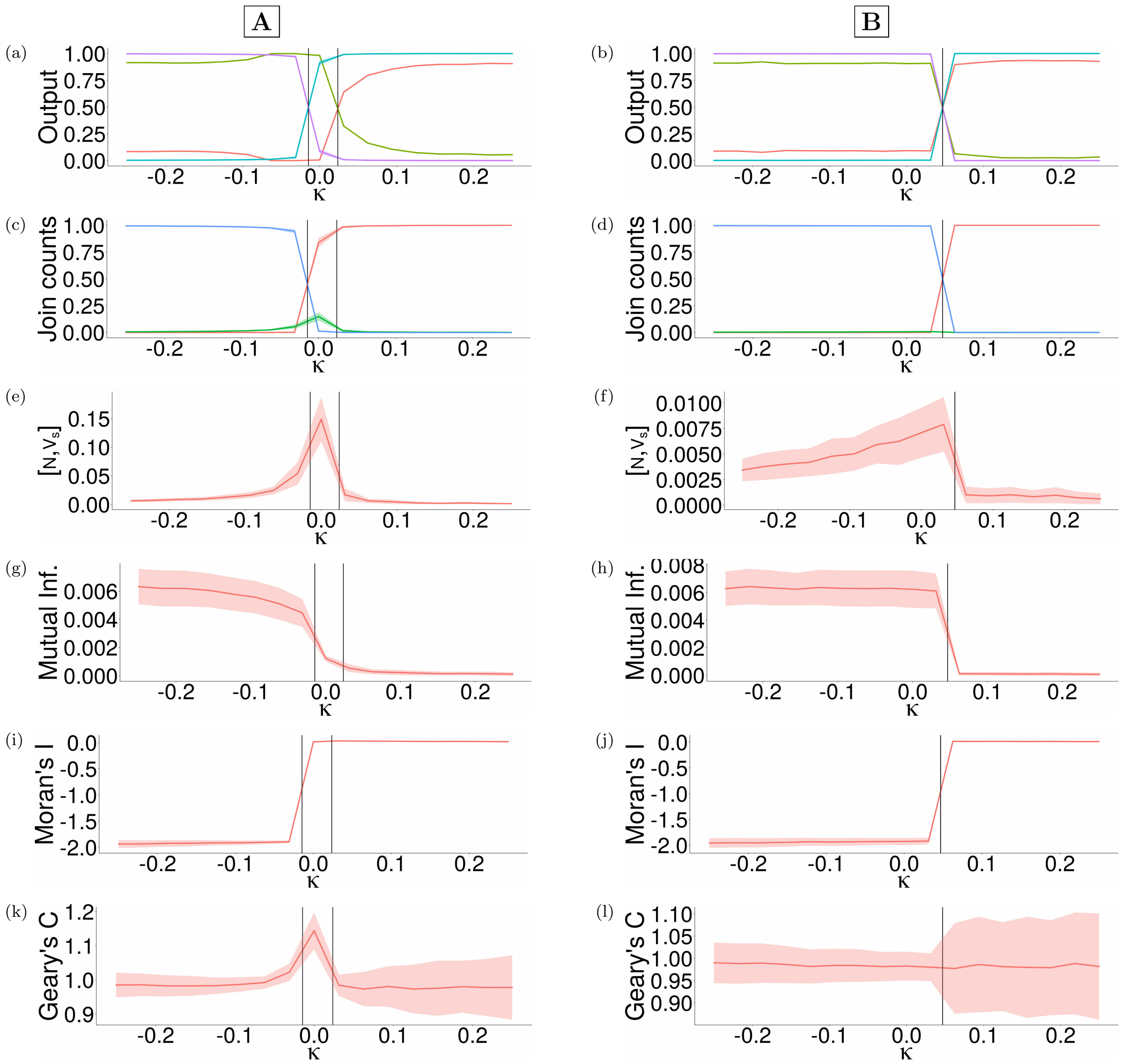


**Figure S7.** Demonstration of the trends of the EWS of model V1 with infectivity $p = 0.8$ approaching the transitions of the social and physical dynamics $K_s$ and $K_p$ (marked by the first and second black lines) respectively, demonstrating the signals given by each tool with respect to the perceived vaccine cost $\kappa$. The intervals in each panel represent one standard deviation of the mean equilibrium value in each stochastic realisation of the model. Social norm $\sigma = 0$ for **(column A)**, and $\sigma = 0.25$ for **(column B)**. **(a-b)** Social dynamics $\langle V_s \rangle$ (green, solid), $\langle N \rangle$ (red, solid) and physical dynamics $\langle R \rangle$ (black, dashed), $\langle V_p \rangle$ (blue, dashed). **(c-d)** Join counts: $\langle N,N \rangle$ (blue), $\langle N,V_s \rangle$ (red), $\langle V_s,V_s \rangle$ (green). **(e-f)** $\langle N,V_s \rangle$ alone. **(g-h)** Mutual information $\langle \mathscr{M} \rangle$. **(i-j)** Moran's I $\langle \mathscr{I} \rangle$. **(k-l)** Geary's C $\langle \mathscr{C} \rangle$.

Supplementary Fig. S16 shows the skewness of the $\kappa$-series of the EWS with respect to the social norm $\sigma$ for models V1 with $p = 0.2$ **(column A)**, $p = 0.8$ **(column B)** and V2 **(column C)**. This quantification of the change of the shape of the $\kappa$-series with increasing $\sigma$ suggests a concern of directionality in the application and interpretation of the EWS; there is no hint that these EWS would be of comparable effectiveness if the model had dynamics such that the derived $\kappa$-series were reversed.

### S5.6 Further comparisons of the EWS' performance

We use the same definitions and measures of performance $\chi^*_{\text{min}}$ maximin and $\chi^*_{\text{max}}$ (maximax) given in the Results section. Figure 6 shows the results of maximax and maximin comparisons of the performances of the EWS and S/P classes of signals using the Lanzante change point detection test for model V2. Supplementary Fig. S17 shows this comparison using all various change point detection tests (Buishand, Lanzante, Pettitt and Standard normal homogeneity tests), and Supp. Fig. S18 makes these comparisons for model V1. Points falling in the green-shaded regions represent $\sigma$ values at which either the

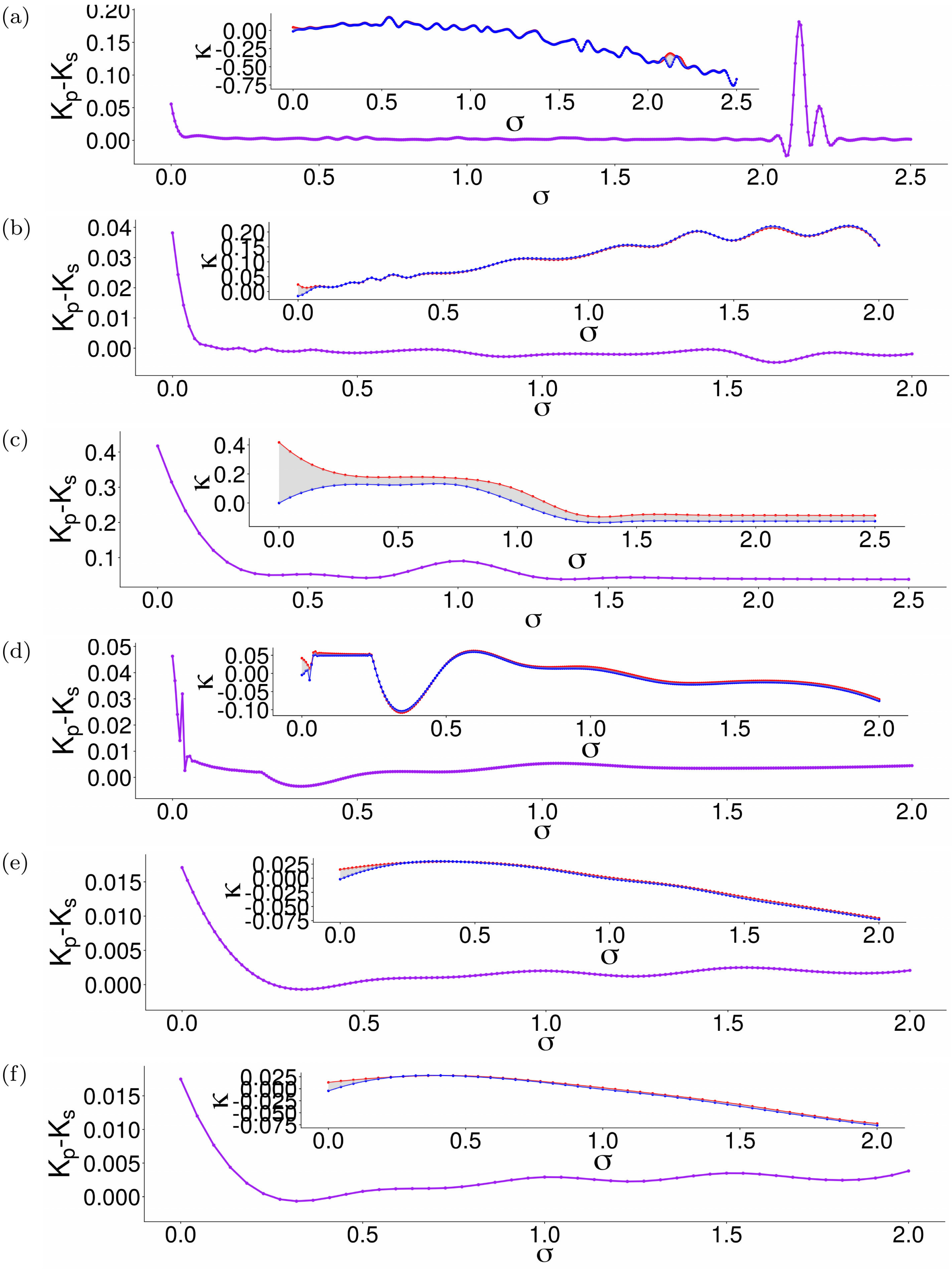


**Figure S8.** Demonstration of the vanishing intertransition distance $K_p - K_s$ (purple) for all models, with inset graphs showing the estimated locations of $K_s$ **(insets, blue)** and $K_s$ **(insets, red)**. **(a)** V1, $p = 0.2$. **(b)** V1, $p = 0.8$. **(c)** V3, $\xi_{52} = 0.1$. **(d)** V3, $\xi_{52} = 0.01$. **(e)** V3, $\xi_{52} = 0.001$. **(f)** V3, $\xi_{52} = 1 \times 10^{-4}$.

best-performing (largest lead time) EWS outperforms all model variables ($\chi^*_{\max} > \varepsilon^*_{\max}$), or where the worst-performing EWS still outperforms the worst-performing variable ($\chi^*_{\min} > \varepsilon^*_{\min}$). Points in the red-shaded region represent the reverse.

Both Supp. Figs. S17 and S18 show equal performance of the EWS and the model variables in both maximin and maximax comparisons, though all panels of Supp. Fig. S18 together show many more instances of equal maximum lead times ($|\chi^*_{\max}| < \varepsilon^*_{\max}$, red points and curve) among the two classes in model V1 than in V2 (Supp. Fig. S17). Also important is the changes in area of the green- and red-shaded regions among the panels of Supp. Figs. S18 and S17; for instance, a large green-shaded region (such as in Supp. Fig. S18g) shows that the maximum lead time of the EWS is much larger than that of the model variables for those $\sigma$ values for which the EWS perform better.

## Summation

In sum, the analysis and comparison of the results of these models allow the conclusion that the behaviours of the EWS are largely independent of the the parameters that differed between the models. Our main results were the reconfirmation of $\langle N, V_s \rangle$ and $\langle \mathscr{M} \rangle$ as the leading EWS of the study through their high lead times, a new attention paid to the use of $\langle N, N \rangle$ as an indicator of transition in V1, and the confirmation of vanishing intertransition distance inherent to the model (and a cause for concern, as stated in the Discussion section). However, it appears that individual lead time of each EWS is subject to the size of the network, the change point detection tests used (each pros and cons[32]), and the (method of) application of these tests for determining the exact location of the first warning given by each EWS.

## References


1. Figueredo, G. P., Siebers, P., Owen, M. R., Reps, J. & Aickelin, U. Comparing stochastic differential equations and agent-based modelling and simulation for early-stage cancer. *PLOS ONE* **9**, e95150, DOI: 10.1371/journal.pone.0095150 (2014).
2. Garnett, G. P., Cousens, S., Hallett, T. B., Steketee, R. & Walker, N. Mathematical models in the evaluation of health programmes. *The Lancet* **378**, 515–525 (2011).
3. Dunham, J. B. An agent-based spatially explicit epidemiological model in mason. *J. Artif. Soc. Soc. Simul.* **9** (2005).
4. Orbann, C., Sattenspiel, L., Miller, E. & Dimka, J. Defining epidemics in computer simulation models: How do definitions influence conclusions? *Epidemics* **19**, 24–32 (2017).
5. Kiss, I. Z., Miller, J. C., Simon, P. L. *et al.* Mathematics of epidemics on networks (2017).
6. for Disease Control, C. & (CDC), P. *Introduction to Epidemiology*, chap. 1, 72–80 (U.S. Department of Health and Human Services, Atlanta, GA 30333, 2012), 2012 edn. A self-study course.
7. Severns, P. M., Estep, L. K., Sackett, K. E. & Mundt, C. C. Degree of host susceptibility on the initial disease outbreak influence subsequent epidemic spread. *J. Appl. Ecol.* **51**, 1622–1630, DOI: 10.1111/1365-2664.12326 (2014).
8. Khan, A. S. & Pesik, N. *Forensic Public HealthL Eoidemiological abd Microbiological Investigations for Biosecurity*, 239–256 (Elsevier, 2011), 2 edn.
9. Bartlett, M. Measles periodicity and community size. *J. Royal Stat. Soc. Ser. A* **120**, 48–70, DOI: 10.2307/2342553 (1957).
10. Nåsell, I. A new look at the critical community size for childhood infections. *Theor. Popul. Biol.* **67**, 203–216 (67).
11. Keeling, M. J. & Grenfell, B. Disease extinction and community size: Modeling the persistence of measles. *Science* **275**, 65–67 (1997).
12. Getz, W. M. & Pickering, J. Epidemic models: Thresholds and population regulation. *The Am. Nat.* **121**, 892–898 (1983).
13. Tessone, C., Toral, R., Amengual, P., H.S., W. & San Miguel, M. Naighbourhood models of minority opinion spreading. *Eur. Phys. J.* (2004).
14. Pellis, L., Fergusson, N. & Fraser, C. Threshold parameters for a model of epidemic spread among households and workplaces. *J. Royal Soc. Interface* **6**, 979–987 (2009).
15. Moore, C. & Newman, M. E. J. Epidemics and percolation in small-world networks. *Phys. Rev. E* **61**, 5678 (2000).
16. Poletti, P., Caprile, B., Ajelli, M., Pugliese, A. & Merler, S. Spontaneous behavioural changes in response to epidemics. *J. Theor. Biol.* **260**, DOI: 10.1016/j.jtbi.2009.04.029 (2009).
17. Wu, Q., Fu, X., Small, M. & Zu, X. The impact of awareness on epidemic spreading in networks. *Chaos* **22**, 013101, DOI: 10.1063/1.3673573 (2012).

**18.** Meloni, S. *et al.* Modeling human mobility responses to the large-scale spreading of infectious diseases. *Sci. Reports* **1**, DOI: 10.1038/srep00062 (2011).

**19.** Sahneh, F., Chowdhury, F. N. & Scoglio, C. M. On the existence of a threshold for preventative behavioral responses to suppress epidemic spreading. *Sci. Reports* **2**, DOI: 10.1038/srep00632 (2012).

**20.** Becker, N. Estimation for an epidemic model. *Biometrics* **32**, 769–777 (1976).

**21.** Chowell, G., Viboud, C., Simonsen, L. & Moghadas, S. M. Characterizing the reproduction number of epidemics with early subexponential growth dynamics. *J. Royal Soc. Interface* **13**, 20160659, DOI: 10.1098/rsif.2016.0659 (2016).

**22.** Liu, Q.-c. *et al.* Measurability of the epidemic reproduction number in data-driven contact networks. *PNAS* **115**, 12680–12685, DOI: 10.1073/pnas.1811115115 (2018).

**23.** Trevelyan, B. & Smallman-Raynor, M. The spatial structure of epidemic emergence: geographical aspects of poliomyelitis in north-eastern usa, july-october 1916. *J. Royal Stat. Soc. Ser. A (Statistics Soc.* **168**, 701–722, DOI: 10.1111/j.1467-985X.2005.00372.x (2005).

**24.** Cliff, A., Haggett, P. & Ord, J. *Spatial Aspects of Influenza Eidemics* (Routledge Hegan & Paul, 1997).

**25.** Cliff, A. & Haggett, P. Methods for the Measurement of Epidemic Velocity from Time-Series Data. *Int. J. Epidemiol.* **11**, 82–89, DOI: 10.1093/ije/11.1.82 (1982).

**26.** Buishand, T. A. Tests for detecting a shift in the mean of hydrolgical time series. *J. Hydrol.* **73**, 51–69 (1984).

**27.** Mallakpour, I. & Villarini, G. A simulation study to examine the sensitivity of the pettitt test to detect abrupt changes in mean. *Hydrol. Scineces J.* **61**, 245–254 (2016).

**28.** Pettitt, A. A non-parametric approach to the change-point problem. *Appl. Stat.* **28**, 126–135 (1979).

**29.** Lanzante, J. R. Resistant, robust and non-parametric techniques for the analysis of climate data: Theory and examples, including applications to historical radiosonde station data. *Int. J. Climatol.* **61** (1996).

**30.** Alexandersson, H. A homogeneity test applied to precipitation data. *J. Climatol.* **6**, DOI: 10.1002/joc.3370060607 (1986).

**31.** Alexandersson, H. & Moberg, A. Homogenization of swedish temperature data. part i: Homogeneity test for linear trends. *Int. J. Climatol.* **17** (1986).

**32.** Easterling, D. R. & Paterson, T. C. A new method for detecting undocumented discontinuities in climatological time series. *Int. J. Climatol.* **15**, 369–377 (1995).

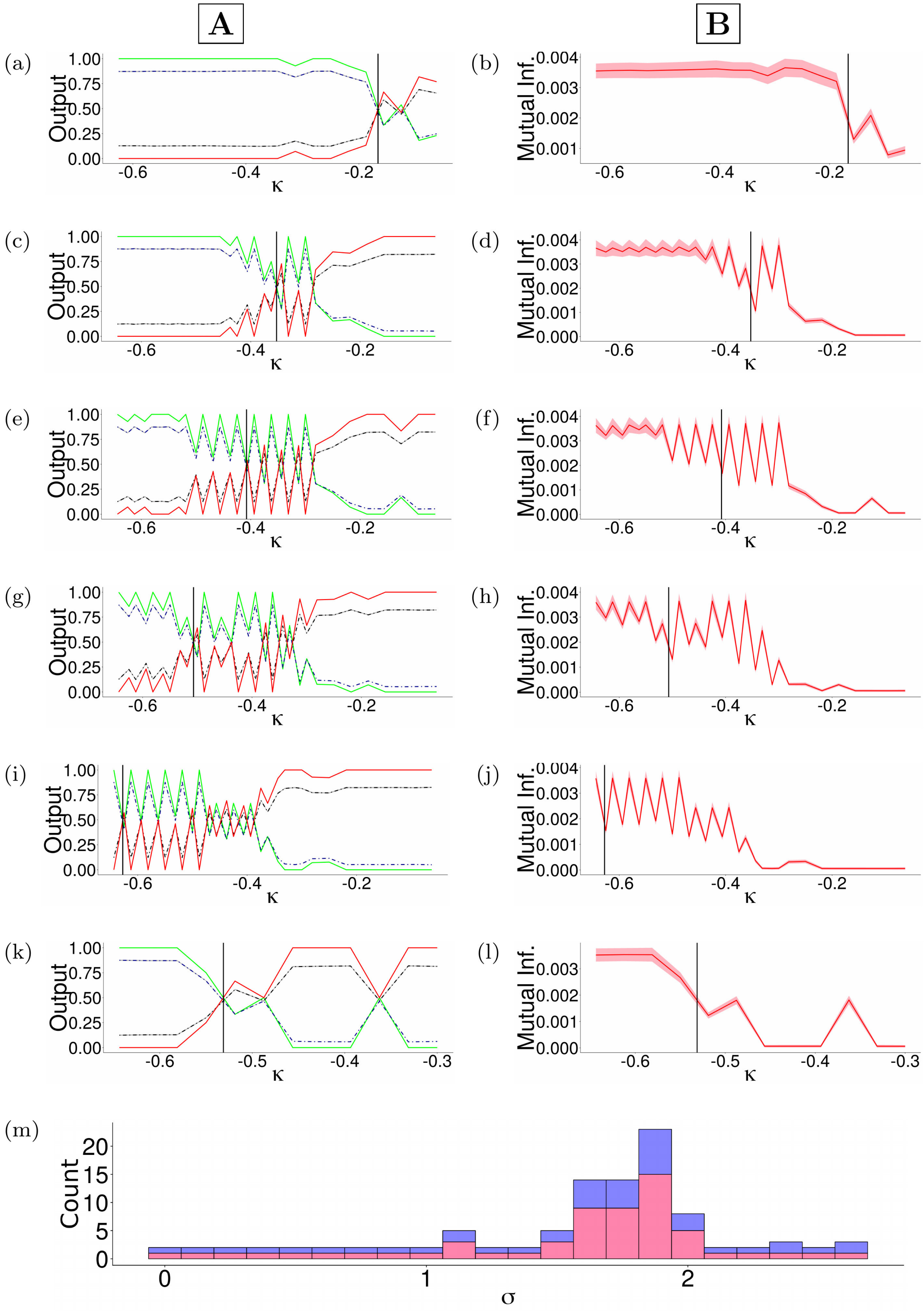


**Figure S9.** For model V2, multiple potential transitions $K_s$ and $K_p$ can be identified for $\sigma > 1$, introducing the opportunity for false warnings unless the earliest possible transition is chosen (in either dynamic), as was done in the main text. This region features erratic behaviour of $\langle \mathscr{M} \rangle$ in regions featuring multiple physical and social transitions. **(Column A)** Social dynamics $\langle V_s \rangle$ (green, solid), $\langle N \rangle$ (red, solid) and physical dynamics $\langle R \rangle$ (black, dashed), $\langle V_p \rangle$ (blue, dashed). **(Column B)** $\langle \mathscr{M} \rangle$ (mutual information, red). **(a-b)** $\sigma = 1.125$. **(c-d)** $\sigma = 1.5$. **(e-f)** $\sigma = 1.625$. **(g-h)** $\sigma = 1.75$. **(i-j)** $\sigma = 2.125$. **(k-l)** $\sigma = 2.25$. The vertical black line (solid) gives the location of $K_s$ and $K_p$. **(m)** gives the number of physical (#$K_p$, red) and social (#$K_s$, blue) transitions with respect to the value of $\sigma$.

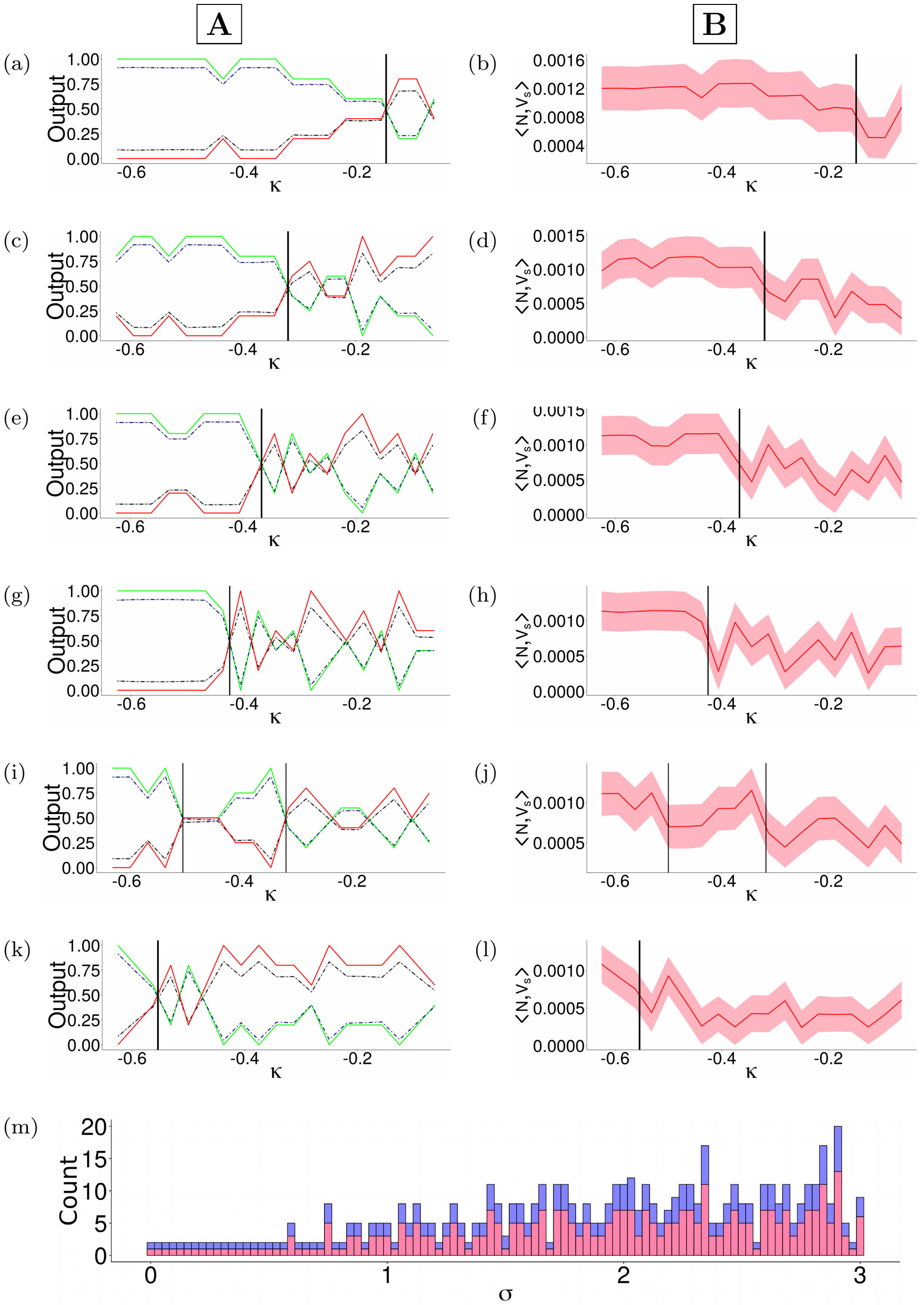


**Figure S10.** Similar to model V2 (Supp. Information S9), multiple social ($\#K_s > 1$) and physical ($\#K_p > 1$) transitions occur in realisations of model V1 ($p = 0.2$) at $\sigma \geq 0.75$, again providing opportunities for false warnings. Erratic behaviour of $\langle N, V_s \rangle$ occurs in regions featuring multiple physical and social transitions. **(Column A)** Social dynamics $\langle V_s \rangle$ (green, solid), $\langle N \rangle$ (red, solid) and physical dynamics $\langle R \rangle$ (black, dashed), $\langle V_p \rangle$ (blue, dashed). **(Column B)** $\langle N, V_s \rangle$ (dissimilar join count, red). **(a-b)** $\sigma = 1.59375$. **(c-d)** $\sigma = 1.8125$. **(e-f)** $\sigma = 1.96875$. **(g-h)** $\sigma = 2.03125$. **(i-j)** $\sigma = 2.125$; this is an instance of multiple social transitions occurring before the first physical transition, presenting an opportunity for the misjudgement of the proximity of a vaccine crisis. **(k-l)** $\sigma = 2.375$. The vertical black line (solid) gives the location of $K_s$ and $K_p$. **(m)** gives the number of physical ($\#K_p$, red) and social ($\#K_s$, blue) transitions with respect to $\sigma$.

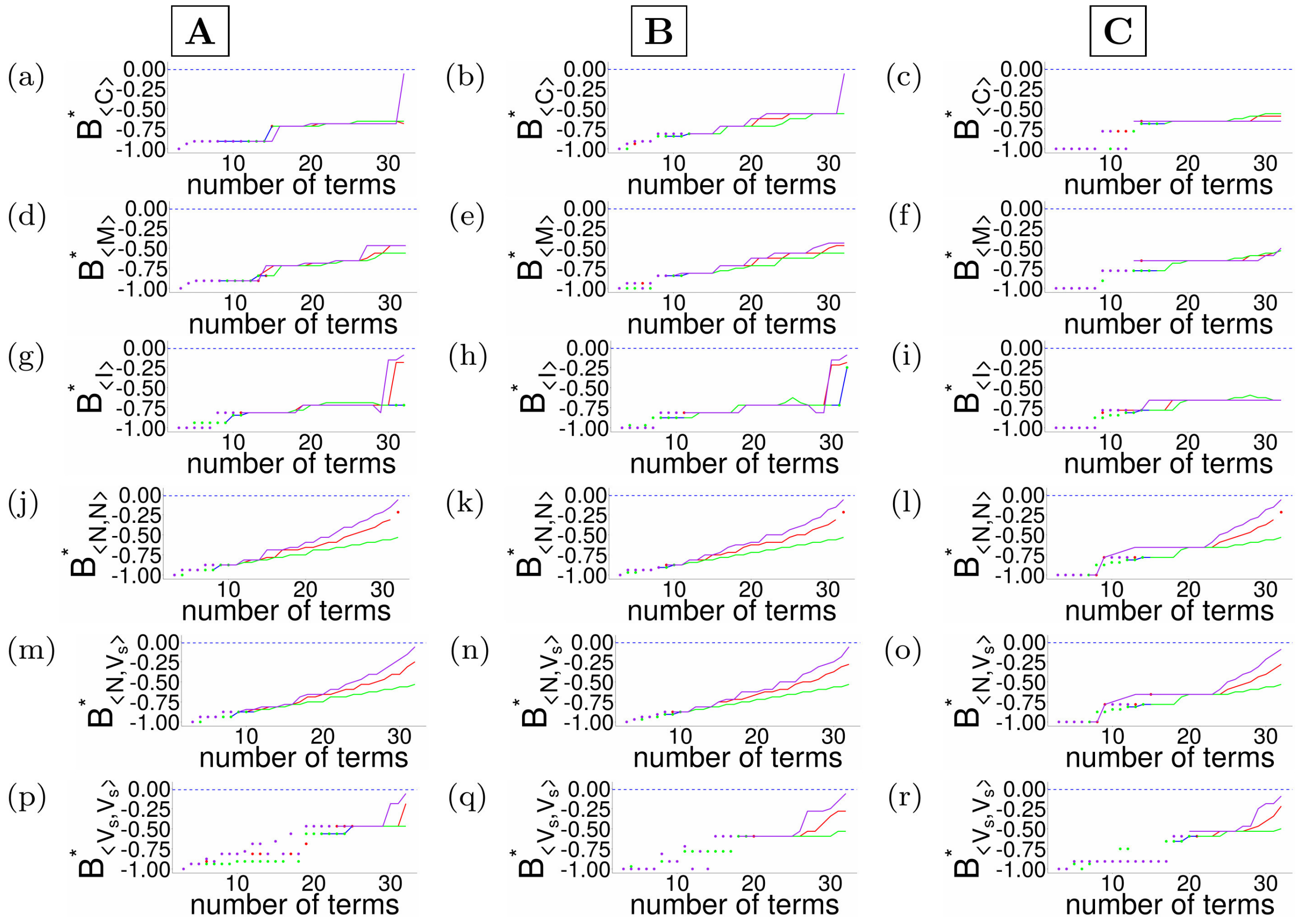

**Figure S11.** Trends of the predicted warnings of the EWS with respect to the length of the $\kappa$-series used. Within each panel, solid lines are estimated change points with $p < 0.05$ (significant), while insignificant estimates ($p \geq 0.05$) are represented by filled points. The Buishand test is represented by red line and points, the Lanzante test by the blue line and points, the Pettitt test by the green line and points, the SNHT by the purple line and points, and the location of $K_s$ is indicated by a blue dashed horizontal line in each panel. **(column A)** model V1 with $p = 0.2$. **(column B)** model V1 with $p = 0.8$. **(column C)** model V2. **(a-c)** the application of the change point tests to the $\kappa$-series for Geary's C $\langle \mathscr{C} \rangle$. **(d-f)** Mutual information $\langle \mathscr{M} \rangle$. **(g-i)** Moran's I $\langle \mathscr{I} \rangle$. **(j-l)** Anti-vaccine similar join count $\langle N, N \rangle$ . **(m-o)** Dissimilar join count $\langle N, V_s \rangle$ . **(p-r)** Pro-vaccine similar join count $\langle V_s, V_s \rangle$ .

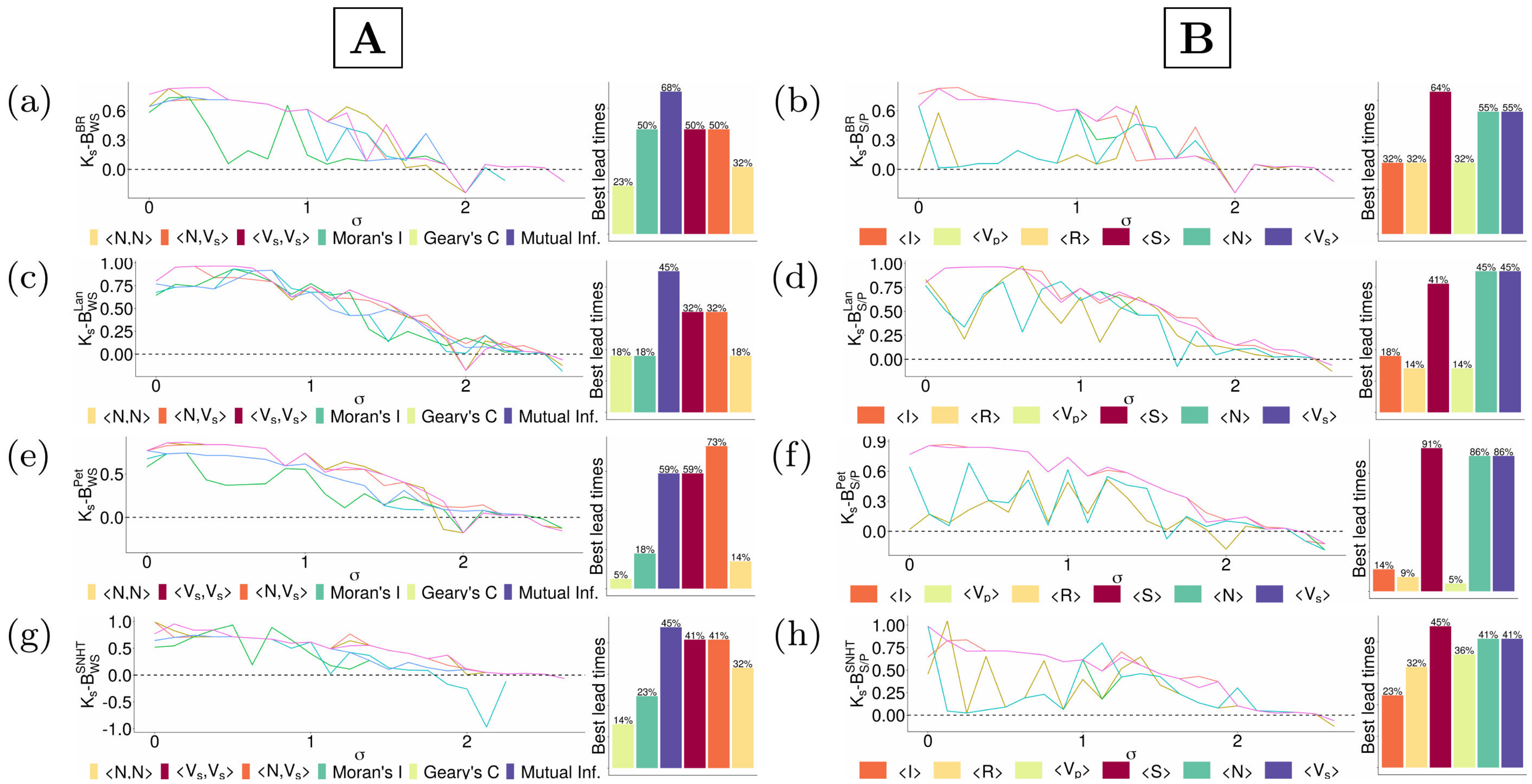


**Figure S12.** The lead times given by different change point detection tests for model V2. The panels of **column (A)** give the results of the tests applied to the early warning signals (EWS), and **column (B)** for the model dynamics. **(a-b)** Buishand range test. **(c-d)** Lanzante test. **(e-f)** Pettitt test. **(g-h)** Standard normal homogeneity test.

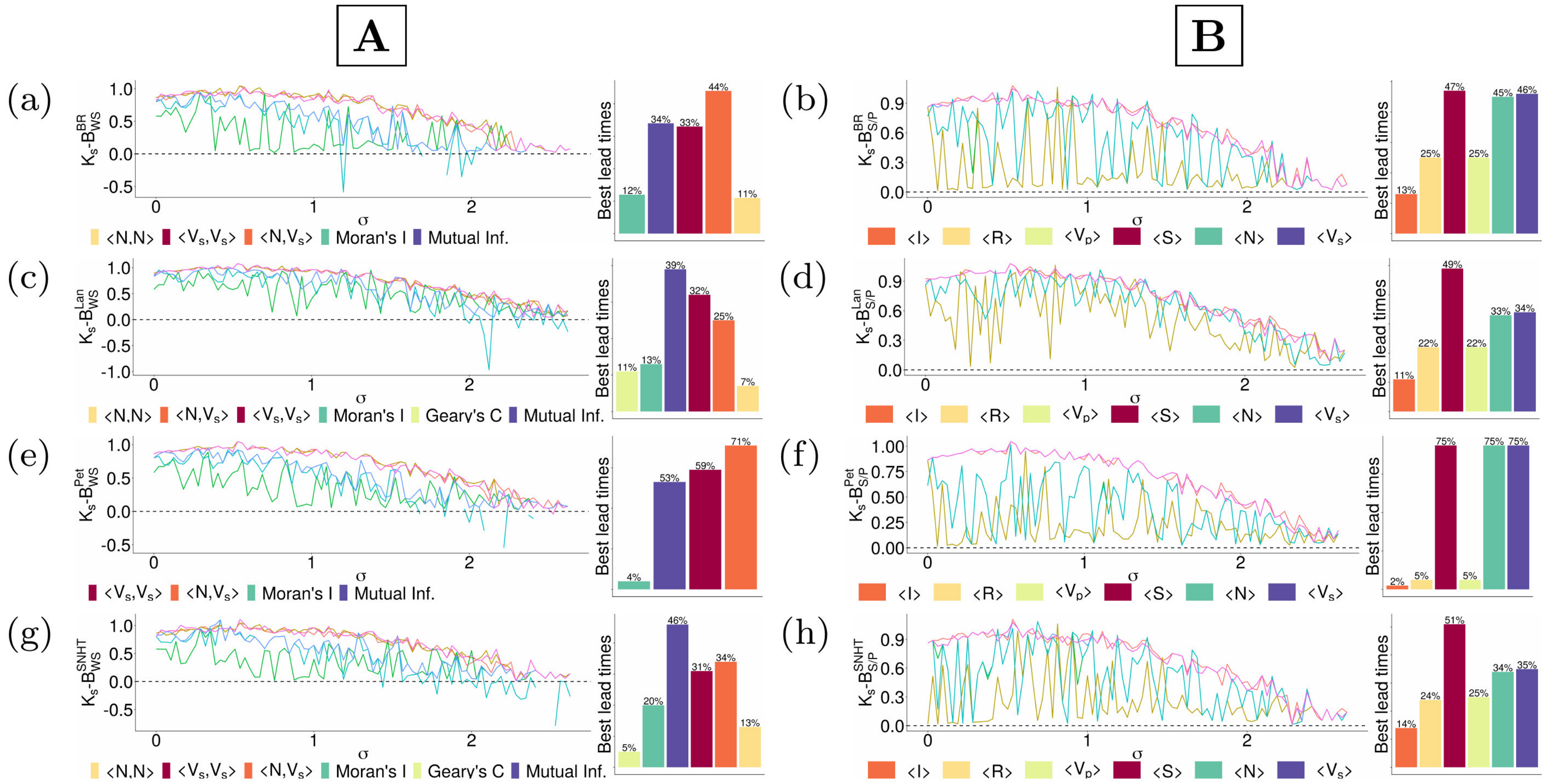


**Figure S13.** The lead times given by different change point detection tests for model V1, with infectivity $p = 0.2$. Similar to Supp. Fig. S12, the panels of **column A** give the results of the tests applied to the early warning signals (WS), and **column B** for the model dynamics. **(a-b)** Buishand range test. **(c-d)** Lanzante test. **(e-f)** Pettitt test. **(g-h)** Standard normal homogeneity test.

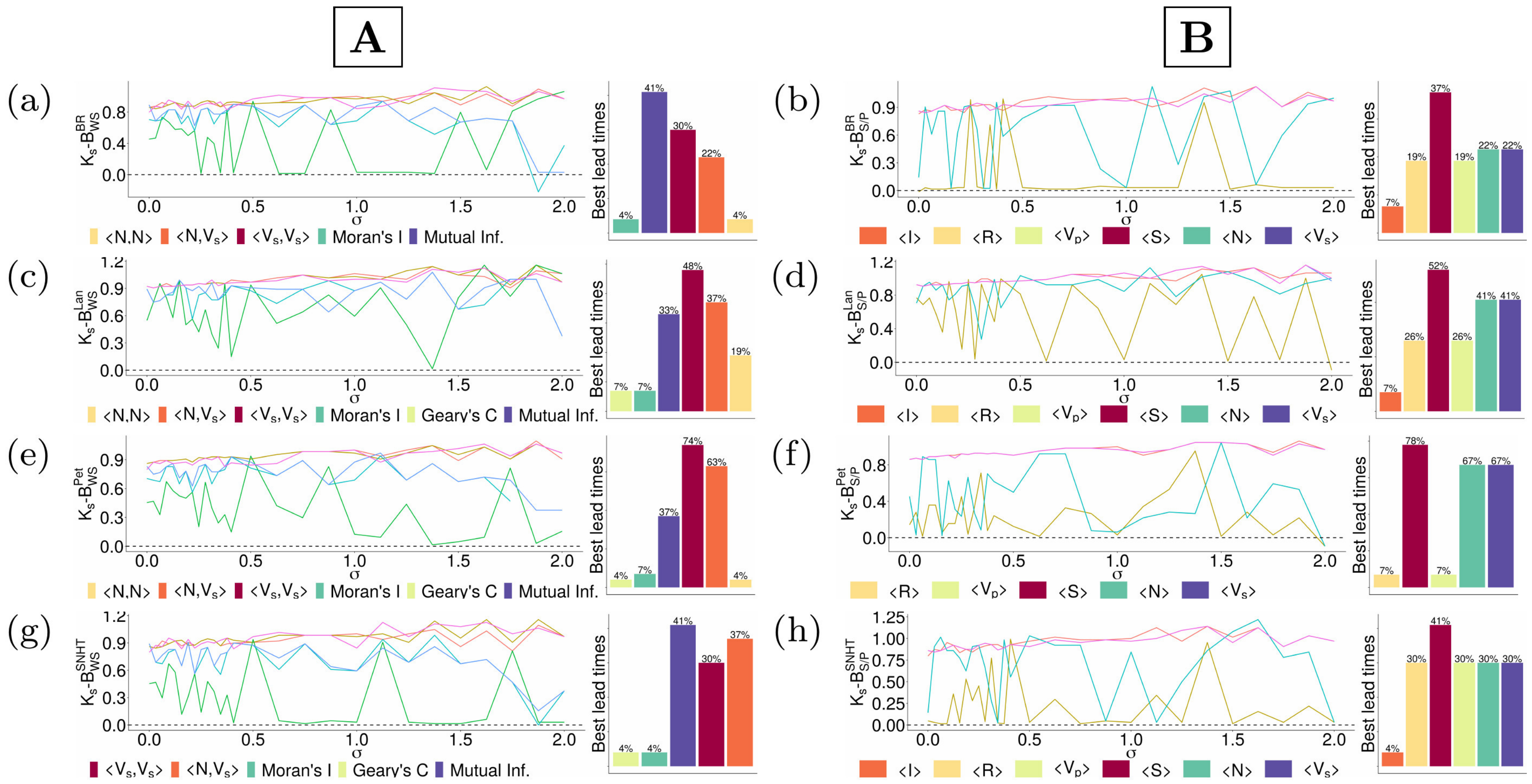


**Figure S14.** The lead times given by different change point detection tests for model V1, with infectivity $p = 0.8$. Similar to Supp. Fig. S13, the panels of **column (A)** give the results of the tests applied to the early warning signals (WS), and **column (B)** for the model dynamics. **(a-b)** Buishand range test. **(c-d)** Lanzante test. **(e-f)** Pettitt test. **(g-h)** Standard normal homogeneity test.

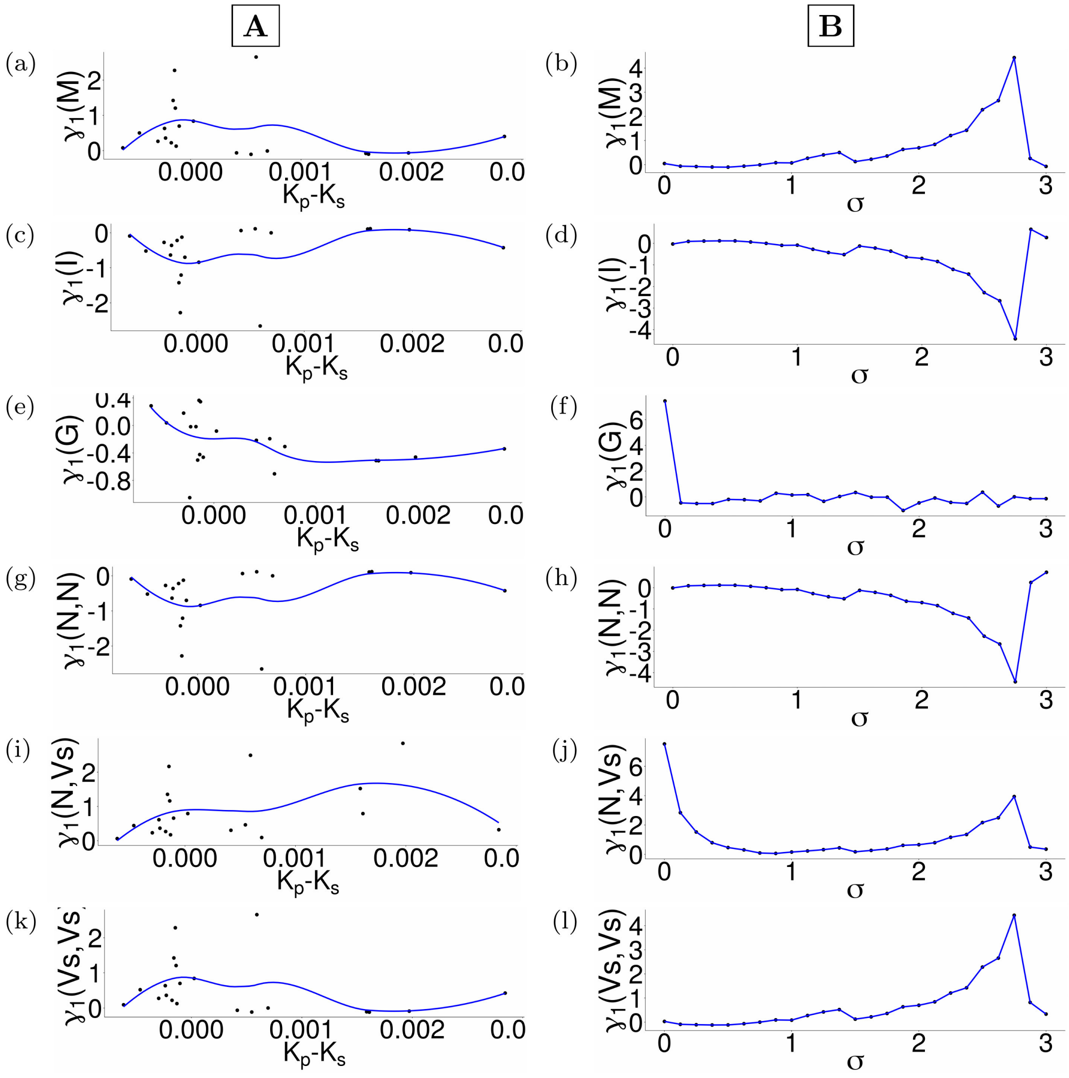


**Figure S15.** The skewness of the EWS' trends varies more consistently with the strength of the social norm $\sigma$ **(column B)** than with the intertransition distance $K_p - K_s$ **(column A)** for model V2. $\kappa$ series such as those shown in Fig. 4, Supp. Figs. S9 and S10 are generally asymmetric about either of the transition ($K_s$ or $K_p$). **(a-b)** Mutual information $\langle \mathscr{M} \rangle$. **(c-d)** Moran's I $\langle \mathscr{I} \rangle$. **(e-f)** Geary's C $\langle \mathscr{C} \rangle$. **(g-h)** $\langle N, N \rangle$ . **(i-j)** $\langle N, V_s \rangle$ . **(k-l)** $\langle V_s, V_s \rangle$ .

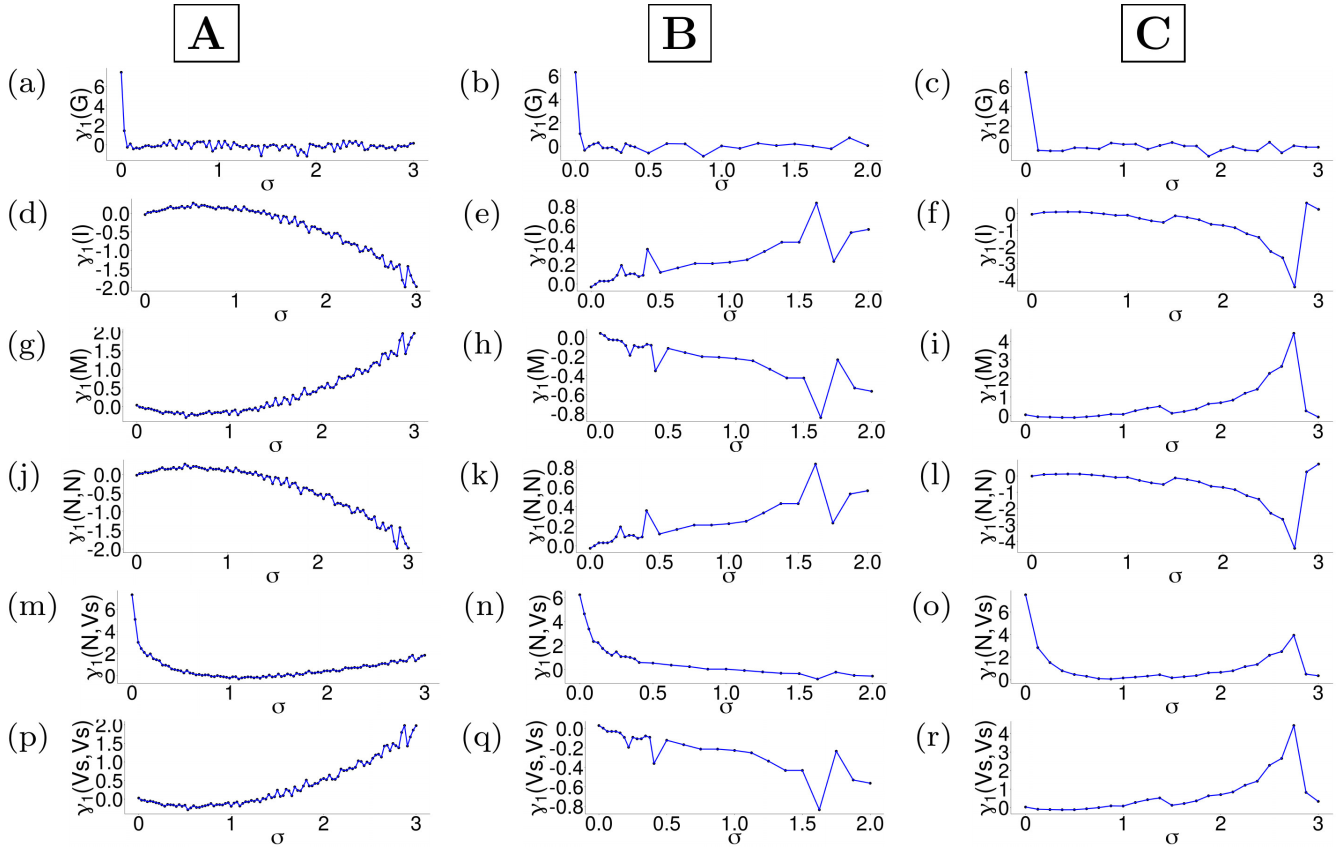


**Figure S16.** Trends of the skewness $\gamma_1$ of the $\kappa$-series with respect to the strength of the social norm $\sigma$ for models V1 with $p = 0.2$ **(column A)**, $p = 0.8$ **(column B)** and V2 **(column C)**. **(a-c)** give the skew of $\langle \mathscr{C} \rangle$, **(d-f)** $\langle \mathscr{I} \rangle$, **(g-i)** $\langle \mathscr{M} \rangle$, **(j-l)** $\langle N,N \rangle$ , **(m-o)** $\langle N,V_s \rangle$ , **(p-r)** $\langle V_s,V_s \rangle$ .

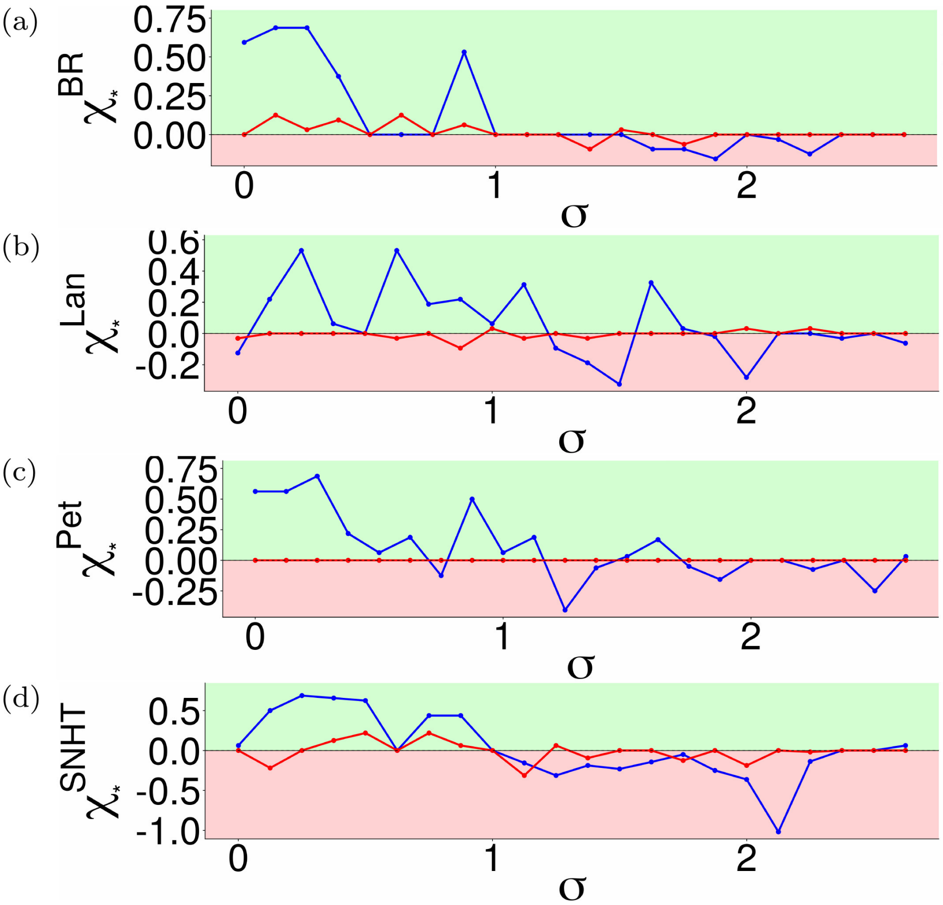


(a)

| | Xmin | Xmax |
|---|---|---|
| neg | 22.7% | 9.1% |
| zero | 54.5% | 63.6% |
| pos | 22.8% | 27.3% |

(b)

| | Xmin | Xmax |
|---|---|---|
| neg | 36.4% | 22.7% |
| zero | 18.2% | 63.6% |
| pos | 45.4% | 13.7% |

(c)

| | Xmin | Xmax |
|---|---|---|
| neg | 31.8% | 0% |
| zero | 13.6% | 100% |
| pos | 54.6% | 0% |

(d)

| | Xmin | Xmax |
|---|---|---|
| neg | 45.5% | 27.3% |
| zero | 18.2% | 50% |
| pos | 36.3% | 22.7% |

**Figure S17.** Comparisons of the performance of EWS (EWS) and model dynamics (S/P) for model V2 with respect to four change point detection tests: **(a)** Buishand range test (BR). **(b)** Lanzante test (Lan). **(c)** Pettitt test (Pet). **(d)** Standard normal homogeneity test (SNHT). In each panel, $\chi^*_{\mathrm{min}}$ is represented by a blue curve through blue points, and $\chi^*_{\mathrm{max}}$ by a red curve through red points. The green-shaded region represents the region where either the worst-performing EWS (that is, giving the smallest lead time) still outperforms with worst-performing model variable ($\chi^*_{\mathrm{min}} > \varepsilon^*_{\mathrm{min}}$) under the specific change point test, or where the best-performing EWS outperforms the best-performing model variable ($\chi^*_{\mathrm{max}} > \varepsilon^*_{\mathrm{max}}$). The inset table gives the ratios of social norm $\sigma$ values for which $\chi^*_* < -\varepsilon^*_*$ (neg), $|\chi^*_*| < \varepsilon^*_*$ (zero) and $\chi^*_* > \varepsilon^*_*$ (pos).

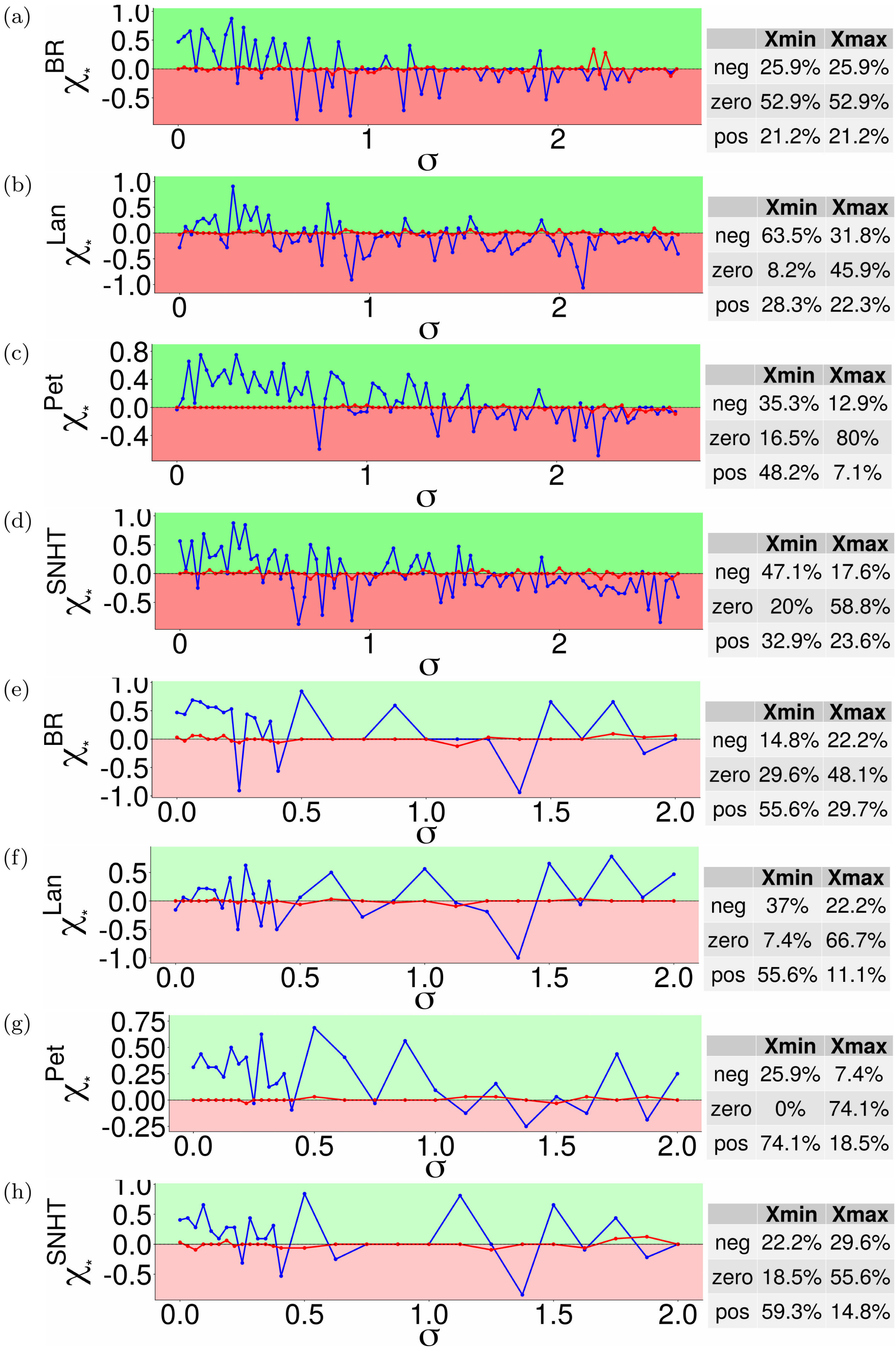


**Figure S18.** Similar to Supp. Fig. S17, we show comparisons of the performance of EWS (WS) and model dynamics (S/P) for model V1 with respect to four change point detection tests: **(a,e)** Buishand range test (BR). **(b,f)** Lanzante test (Lan). **(c,g)** Pettitt test (Pet). **(d,h)** Standard normal homogeneity test (SNHT). **(a-d)** infectivity $p = 0.2$, **(e-h)** infectivity $p = 0.8$. In each panel, $\chi^*_{\min}$ is represented by a blue curve through blue points, and $\chi^*_{\max}$ by a red curve through red points. The green-shaded region represents the region where either the worst-performing EWS (that is, giving the smallest lead time) still outperforms with worst-performing model variable ($\chi^*_{\min} > \varepsilon^*_{\min}$) under the specific change point test, or where the best-performing EWS outperforms the best-performing model variable. The inset table gives the ratios of social norm $\sigma$ values for which $\chi^*_* < -\varepsilon^*_*$ (neg), $|\chi^*_*| < \varepsilon^*_*$ (zero) and $\chi^*_* > \varepsilon^*_*$ (pos).
